# Steiner tree methods for optimal sub-network identification: an empirical study

**DOI:** 10.1186/1471-2105-14-144

**Published:** 2013-04-30

**Authors:** Afshin Sadeghi, Holger Fröhlich

**Affiliations:** 1 Bonn-Aachen International Center for IT, Rheinische Friedrich-Wilhelms Universitat Bonn, Dahlmannstr 2, 53113 Bonn, Germany

## Abstract

**Background:**

Analysis and interpretation of biological networks is one of the primary goals of systems biology. In this context identification of sub-networks connecting sets of seed proteins or seed genes plays a crucial role. Given that no natural node and edge weighting scheme is available retrieval of a minimum size sub-graph leads to the classical Steiner tree problem, which is known to be NP-complete. Many approximate solutions have been published and theoretically analyzed in the computer science literature, but far less is known about their practical performance in the bioinformatics field.

**Results:**

Here we conducted a systematic simulation study of four different approximate and one exact algorithms on a large human protein-protein interaction network with ~14,000 nodes and ~400,000 edges. Moreover, we devised an own algorithm to retrieve a sub-graph of merged Steiner trees. The application of our algorithms was demonstrated for two breast cancer signatures and a sub-network playing a role in male pattern baldness.

**Conclusion:**

We found a modified version of the shortest paths based approximation algorithm by Takahashi and Matsuyama to lead to accurate solutions, while at the same time being several orders of magnitude faster than the exact approach. Our devised algorithm for merged Steiner trees, which is a further development of the Takahashi and Matsuyama algorithm, proved to be useful for small seed lists. All our implemented methods are available in the R-package SteinerNet on CRAN (http://www.r-project.org) and as a supplement to this paper.

## Background

Analysis of biological networks is one of the primary goals of systems biology [1]. Databases, like KEGG [2], HPRD [3] and PathwayCommons [4] nowadays store tens of thousands of literature reported molecular interactions and thus facilitate the interpretation of biological data. One particular aspect within this context is the construction of sub-networks connecting specified seed genes or proteins of biological interest. Whereas an obvious way to address this problem is to enumerate and join all possible shortest paths between the molecules of interest [5,6], this solution is not guaranteed to produce *minimal* sub-networks. Inclusion of large numbers of auxiliary nodes and edges in addition to the ones of primary interest, however, can greatly complicate the visualization and interpretation of the constructed sub-networks.

The task of identifying an optimal sub-network for a given set of seed genes or proteins can be viewed as an instance of the Steiner tree [7] or the Prize Collecting Steiner tree problem [8], depending on whether or not additional weights for nodes (seen as *profits*) and edges (seen as *costs*) are available. Briefly, a Steiner tree is a sub-graph connecting all seed nodes (called *terminals*) within the original molecular interaction network. The Steiner tree problem is to find a Steiner tree of minimal size, i.e. minimal number of edges. This problem is known to be NP-complete [9]. The same holds true for the Prize Collecting Steiner tree (PCST) problem, where the task is to find a Steiner tree with maximal profit at minimal cost.

Several authors have noticed the relationship between optimal sub-network identification in molecular networks and the (Prize Collecting) Steiner tree problem [10-15]. Most of these authors focused on weighted networks, leading to the PCST problem: Scott et al. [10] showed that an approximate PCST algorithm could recover known regulatory interaction networks responding to heat shock in yeast with high accuracy. Dittrich et al. employed an exact approach using integer linear programming to identify disease related sub-networks in cancer [12]. The method has been made publicly available in the Bioconductor R-package BioNet [16]. Tuncbag et al. [17] recently also published a web service for biological network analysis using an exact algorithm for the PCST problem. Bailly-Bechet al. [15] proposed to approximate the PCST problem via belief propagation and applied it successfully to identify sub-networks responding to drug perturbations in yeast. Huang and Fraenkel [11] used an exact PCST algorithm to determine a protein interaction network playing a role in yeast pheromone response.

In this paper we focus on the classical Steiner tree problem in *unweighted* graphs. This problem appears, if people want to query molecular interaction databases with a list of seed proteins in order to get some understanding about their possible interplay, but there is no reasonable node weighting scheme available, because there is no experimental data, which can be mapped on the network, or because the interaction database does not contain edge confidence scores. Most currently available interaction databases (e.g. PathwayCommons, HPRD, KEGG) do not contain edge confidence values. An important difference of the classical to the PCST problem is that in a PCST solution there is no guarantee that all seed nodes are included. In contrast, in the classical Steiner tree problem that is the case. The classical Steiner tree problem is hence more natural for sub-network identification in the lack of any suitable node weighting scheme.

Molecular interaction networks can be quite large-we here used a human protein interaction network with more than ~13,000 nodes and ~400,000 edges. Thus exact algorithms can become quickly impractical, and hence approximate solutions are of high interest. Whereas in theoretical computer science the Steiner tree problem is principally well studied, there is little known about the practical performance of heuristic algorithms in real biological networks. We thus set up a simulation study to address the following questions:

1. How accurate are our tested heuristic methods compared to an exact solution?

2. How do these heuristics compare to each other in terms of the solution quality and run time?

3. How can we determine several solutions of equal quality/size in an efficient way?

The last questions attributes the fact that the solution to the Steiner tree problem is not necessarily unique (see Methods part for an example). That means there can principally exist several Steiner trees of equal size, and without any further knowledge there is absolutely no reason to prefer one over another solution. To our best knowledge the problem of multiple solutions is not well studied. Besides an exact solution we here propose a straight forward extension of the shortest paths heuristic by Takahashi and Matsuyama [18] for this purpose.

After extensive simulation studies we investigated three real life scenarios: First, the interplay between androgen receptor (*AR*) and *HDAC*9 in the context of male pattern baldness [19]. Second, the well-known 70-gene signature for breast cancer prognosis by van't Veer et al. [20], and third, the 286-gene invasive breast cancer signature by Wang et al. [21]. We show that the (extended) shortest paths heuristic provides clearly interpretable results in all three cases.

## Methods

### Protein-protein interaction network

A protein interaction network was compiled from a merger of all non-metabolic KEGG pathways [2]. Only gene nodes were considered together with the Pathway Commons database [4], which was downloaded in tab-delimited format (May 2010). The purpose was to obtain an as much as possible comprehensive network of known protein interactions. For the Pathway Commons database the SIF interactions INTERACTS WITH and STATE CHANGE were taken into account^a^ and any self loops removed. For retrieval and merger of KEGG pathways, we employed the R-package KEGGgraph [22]. All edge directions were ignored, resulting in a network graph of 13,840 nodes and 397,454 edges. Nodes in this network were identified via Entrez gene IDs. The largest connected component of this graph, which we considered for the following had 13,340 nodes and 397,366 edges.

### Problem formulation

The Steiner tree problem is defined as follows [7]: Given a graph *G* = (*V*, *E*) and a set of terminal nodes *S* ⊆ *V* find a sub-graph *G*′ = (*V*′, *E*′) of *G*, such that

1. *S* ⊆ *V*′ ⊆ *V and E*′ ⊆ *E*

2. There exists a path between every pair of terminals in *G*′.

The set of auxiliary nodes *N* := *V*′ \ *S* is called *non-terminals*. A Steiner tree is called minimal if |*E*′| is minimal.

### Exact algorithm

Multiple approaches have been devised to retrieve exact solutions for the Steiner tree problem. All these algorithms have a run time, which is scaling exponentially with the number of nodes in the graph and are hence difficult to scale up to larger biological networks. They investigate all possible Steiner trees in order to find a minimal one. Examples of exact algorithms include the one by Lawler [23], which works on distance networks, the one by Balakrishnan and Patel [24], which is formulated as a degree constrained sub-graph problem, and the dynamic programming approaches by Dreyfus [25] and Levin [26]. Also branch and bound approaches [27-30] and linear programming solutions have been devised [31].

Here, we implemented a minimal spanning tree based algorithm, which has been described by Hakimi [32]. Briefly, the idea is to investigate all possible subsets of nodes, which include all |*S*| terminals. If there exists a minimum spanning tree between these nodes, then this tree is a candidate solution for the Steiner tree problem. By exhaustively searching through all possible subsets we are guaranteed to find the optimal solution. There are $\sum_{i=o}^{\left|S\right|-2}\left(\begin{array}{l} V-\left|S\right|\\ \;i \end{array}\right)\;\leq \;2^{V-\left|S\right|}$ minimum spanning trees to be determined in this approach. It has to be noted here that in case of an unweighted graph every spanning tree is also a minimum spanning tree. Every spanning tree over n nodes contains exactly n-1 edges. Hence it is sufficient to test for each candidate subset *A* of nodes, whether they form a connected sub-graph of *G* of size n - 1*.* Testing, whether nodes *A* form a connected sub-graph can be done in linear time via a depth first or a breadth first search [7].

### Shortest paths based approximation (SP)

**Figure 1:**
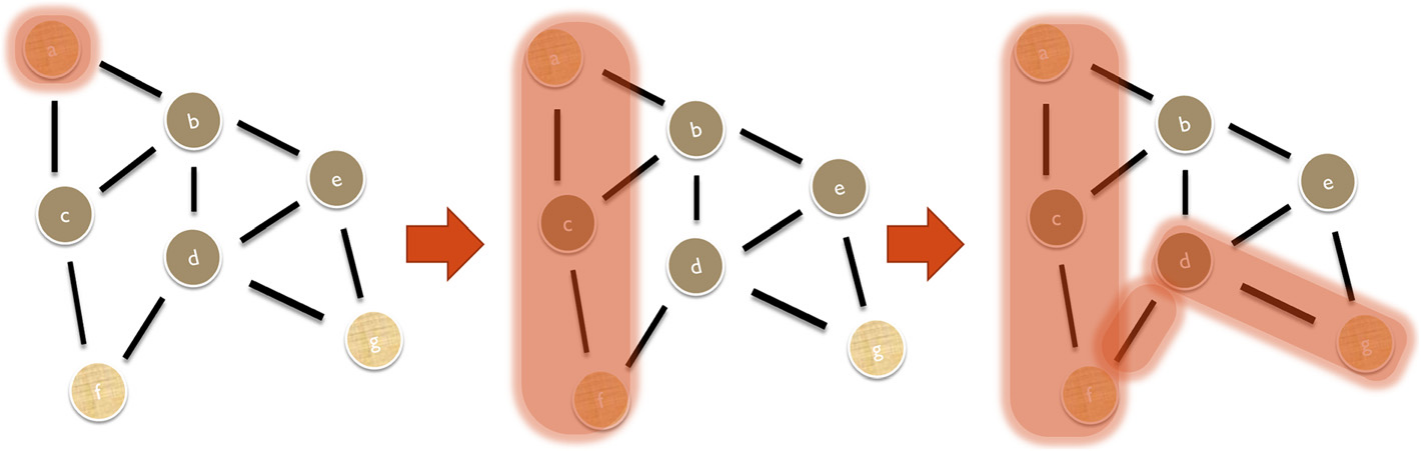
**Illustration of the shortest paths heuristic: The algorithm starts with an arbitrarily picked terminal *a *(left).** Then the terminal (*f*) closest to *a* is picked and the shortest paths connecting *a* to *f* added to the temporary Steiner tree *G*′ (middle). The algorithm proceeds by finding the terminal (*g*) closest to all nodes in *G*’. Accordingly, the shortest path from *f* to *g* is added to *G*′ (right).

A relatively simple but effective [33] heuristic approach to obtain an approximate solution for the Steiner tree problem is based on shortest path computations between terminals. Takahashi and Matsuyama [18] suggested this method in 1980 and proved the size of the resulting Steiner tree to be upper bounded by 2 − 2/|*S*| times the size of the minimal Steiner tree. The algorithm starts with one arbitrarily picked terminal *s*. Then it selects a terminal node t with shortest path distance to s (note that there could be several ones). The shortest path from *s* to *t* (including s itself) is now regarded as a sub-graph *G*′ *of G*. The algorithm proceeds by finding a terminal node *k* with shortest path distance to all nodes in *G* and merging the corresponding path with *G*′ (Figure 1). This step is repeated until all terminals have been included into *G*′. Rayward-Smith and Clare [34] improved this algorithm further by returning the minimum spanning tree on *G*′ and removing all non-terminal nodes of degree one.

The result of the shortest path method depends on the selected start node. Winter and Smith [35] thus suggested to repetitively construct Steiner trees with different randomly chosen start nodes (here: 10). The whole algorithm is shown in pseudo-code 1. The computationally bottleneck is the computation of pairwise shortest path distances in an unweighted graph (line 7). Using a depth- first search this step has a complexity of *O*(|*V*| + |*E*|) [7], because we can look for all terminals not in *G*′ at once. Hence the whole loop is of *O*(*r*|*S*|(|*V*| + |*E*|)). The number r of repeats was set to 10 here.

### Minimum spanning tree based approximation (KB)

A second heuristic approach, which we tested here, has a certain similarity to Kruskal's minimum spanning tree algorithm [36] and is described by Wang [37]. The algorithm starts by initially considering each terminal as a separate graph. Then sequentially those sub-graphs are merged, which are closest to each other. The distance of two sub-graphs $f_{{}_{i}},f_{{}_{j}}$ is measured by the minimal shortest path distance between any pair of nodes in *f*_*i*_ and *f*_*j*_. Pseudo-code 2 shows the algorithm called Kruskal-Based heuristic here. In our implementation we added the two optimization steps by Rayward et al. [34] described previously for the shortest path heuristic. The size of the KB constructed Steiner tree is at most 2–2/|*l*| times the size of the minimal Steiner tree, where *l* is the number of leaves in the minimal Steiner tree [38,39]. The computational bottleneck is line 5. Using again a depth or breadth first search strategy the necessary shortest path computations can be done in *O*(|*V*| + |*E*|) per individual *f*_*i*_. Hence line 5 takes *O*(|*S*|(|*V*| + |*E*|)). Furthermore, in the worst case the loop has to be executed |*S*| times until all terminals appear in one sub-graph *f*_*i*_. Therefore, the whole algorithm has time complexity *O*(|*S*|^2^(|*V*| + |*E*|)), which is inferior to the SP method, if *r* < |*S*|.

### Randomized all shortest paths approximation (RSP)

In addition to the afore mentioned two heuristic methods we experimented with an own approach, which is a randomized algorithm. The idea is to start with the sub-graph G* consisting of all nodes and edges appearing on shortest paths between terminals. A minimum spanning tree *T* is constructed on G*. Afterwards randomly a non-terminal node *ν* ∈ G* is selected and removed from G*, unless G* would fall into two connected components. Then a minimum spanning tree over the remaining sub-graph G* \ {*v*} is constructed. If this spanning tree has a smaller size than *T*, the removal of the node is accepted, otherwise rejected. Similarly the algorithm tests, whether the insertion of randomly picked non-terminals from the complement graph *G* \ G* would decrease the size of the minimum spanning tree. The whole procedure is repeated *r* times (here: 70) and shown in pseudo-code 3. We also tried larger values of *r* without observing significant differences to the results presented here.

In each loop the algorithm has to construct a minimum spanning tree, which can be done in *O*(|*E*| *log* |*V*|) time via Kruskal's algorithm [7]. Determination of all terminals within *max_len* distance can be performed in *O*(|*V*| + |*E*|) time via a breadth- first search. Hence, the loop costs *O*(|*E*| *log* |*V*| + |*V*| + |*E*|) time.

Pre-computation of the all shortest paths between terminals (line 3) is doable in *O*(|*S*|(|*V*| + |*E*|)) (see above). Therefore, the overall computational cost of the algorithm is *O*((*r* + |*S*|)(|*V*| + |*E*|) + *r*|*E*| *log*(|*V*|)).

### All shortest paths between terminals (ASP)

For comparison reasons we also included a trivial method, in which a sub-graph consisting of pairwise shortest paths between terminals was computed. The run time complexity for finding all pairwise shortest paths is *O*(|*S*|(|*V*| + |*E*|)), as described above. Merging all paths (lists of node sequences for each terminal) into one graph additionally requires *O*(|*S*|^2^|*V*|) edge insertions. Such a step can be circumvented in the implementation of the other algorithms.

### Sub-graph of merged minimal steiner trees

**Figure 2:**
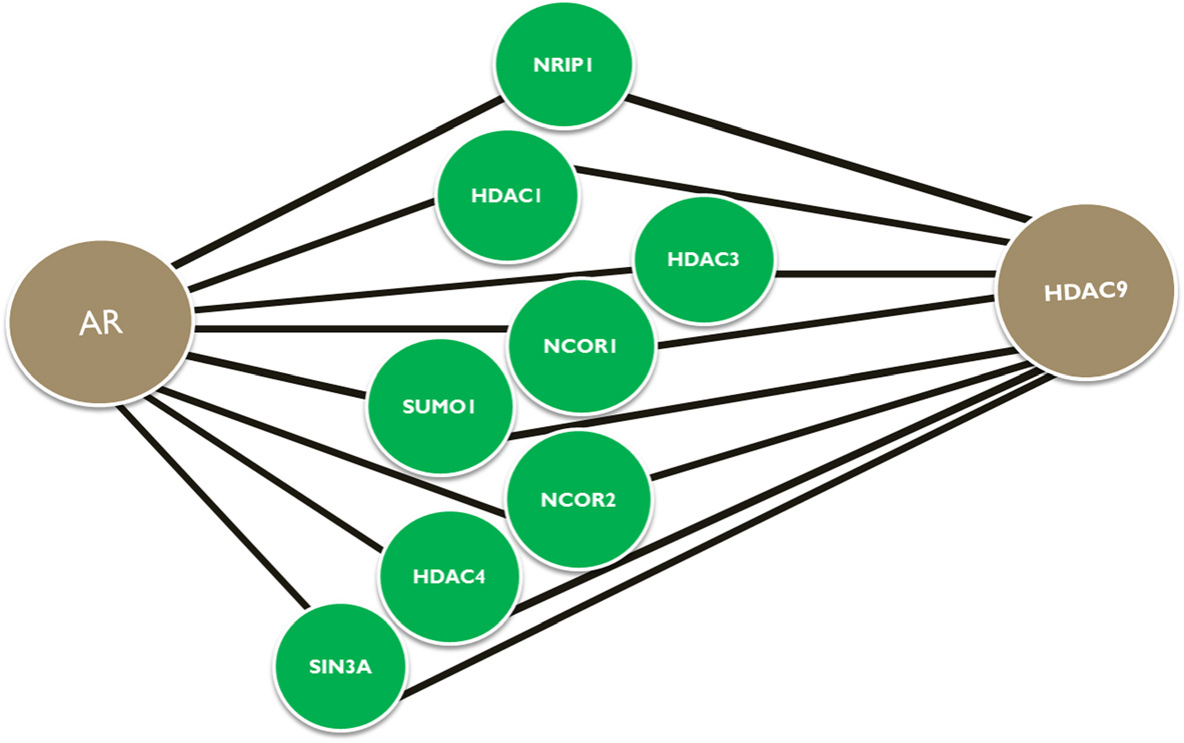
***AR - HDAC9 *interaction network.**

For a given Steiner tree problem there can principally exist several solutions of the same size. As an example consider the graph in Figure 2. Suppose *AR* and *HDAC*9 are the two terminals, then obviously any of the Steiner trees *AR*-*NRPI*1-*HDAC*9, *AR*-*HDAC*1-*HDAC*9, *AR*-*HDAC*3-*HDAC*9, *AR*-*NCOR*1-*HDAC*9, *AR*-*SUMO*1-*HDAC*9, *AR*-*NCOR*12-*HDAC*9 and *AR*-*HDAC*4-*HDAC*9 have exactly the same minimal size. From a biological point of view and without any further information there is absolutely no reason to prefer one of these Steiner trees over another one.

Hence, we should consider all possible solutions of the same size as equally relevant.

Taking this into account we here implemented a modification of the exact algorithm, in which all Steiner trees of minimal size are returned. Obviously the worst-time run time complexity is unaffected by this modification. Afterwards all Steiner trees are merged to one sub-graph, which is then further considered.

### Sub-graph of merged steiner trees (STM)

Since the exact algorithm has an exponential run time complexity and is therefore limited in its practical applicability we also implemented a straight forward modification of the shortest path heuristic (Pseudo-code 1) for computing a sub-graph containing several Steiner trees of possibly same size: Instead of arbitrarily picking one of the terminals with equal shortest path distance to all nodes in sub-graph *G*′ we select all (line 7). Then we join all possible shortest paths to these nodes to *G*′ (line 8). The sub-graph *G*′ at the end will thus contain a merger of several Steiner trees.

### Performance measures

Let *G*′ = (*V*, *E*) be the Steiner tree sub-graph constructed by one of our tested algorithms and *S* the set of terminal nodes. We evaluated the quality of a solution based on the following two criteria:

•Number of edges, |*E*| of the Steiner tree

•Terminal frequency, defined as

$$\frac{\left|S\right|}{\left|V\right|}$$

In addition we looked at the raw computation time, which was determined on an 8 core Intel Xeon system with 2.8 GHz and 90GB RAM.

## Results

### Simulations

#### Experimental setup

To simulate the behavior of each of our tested algorithms (4 heuristic, one exact) we compiled a large protein-protein interaction for human comprising ~13,000 nodes and ~400,000 edges (see Methods). In order to generate seed lists of proteins within this network, we randomly picked a start node and then conducted a random walk, in which with a given probability *θ* = 0.5 each visited node was declared to be a terminal. The random walk was terminated once a predefined number of terminals had been collected. This process was repeated 50 times, and each time our tested algorithms were asked to construct the Steiner tree.

### Shortest paths based approximation outperforms other heuristics

We first addressed the question, which of our four tested heuristics (SP, KB, RSP, ASP) performed best with respect to the size and the terminal frequency of reconstructed Steiner trees (see Methods part for definitions). This was done for terminal sets with 5, 8, 20, 50, 70 and 150 proteins.

**Figure 3:**
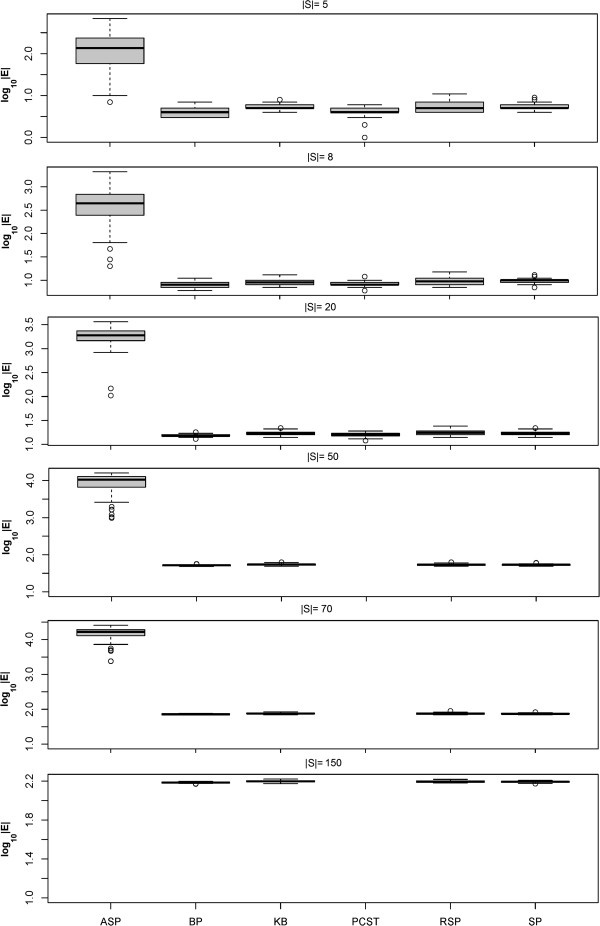
**Size (|*E*|) of constructed sub**-**networks with different heuristic Steiner tree algorithms**, **the exact PCST method and the belief propagation approximation (BP).** For |*E*| ≥ 50 the PCST algorithm became impractical slow. The same was true for the ASP algorithm for |*E*| = 150.

**Figure 4:**
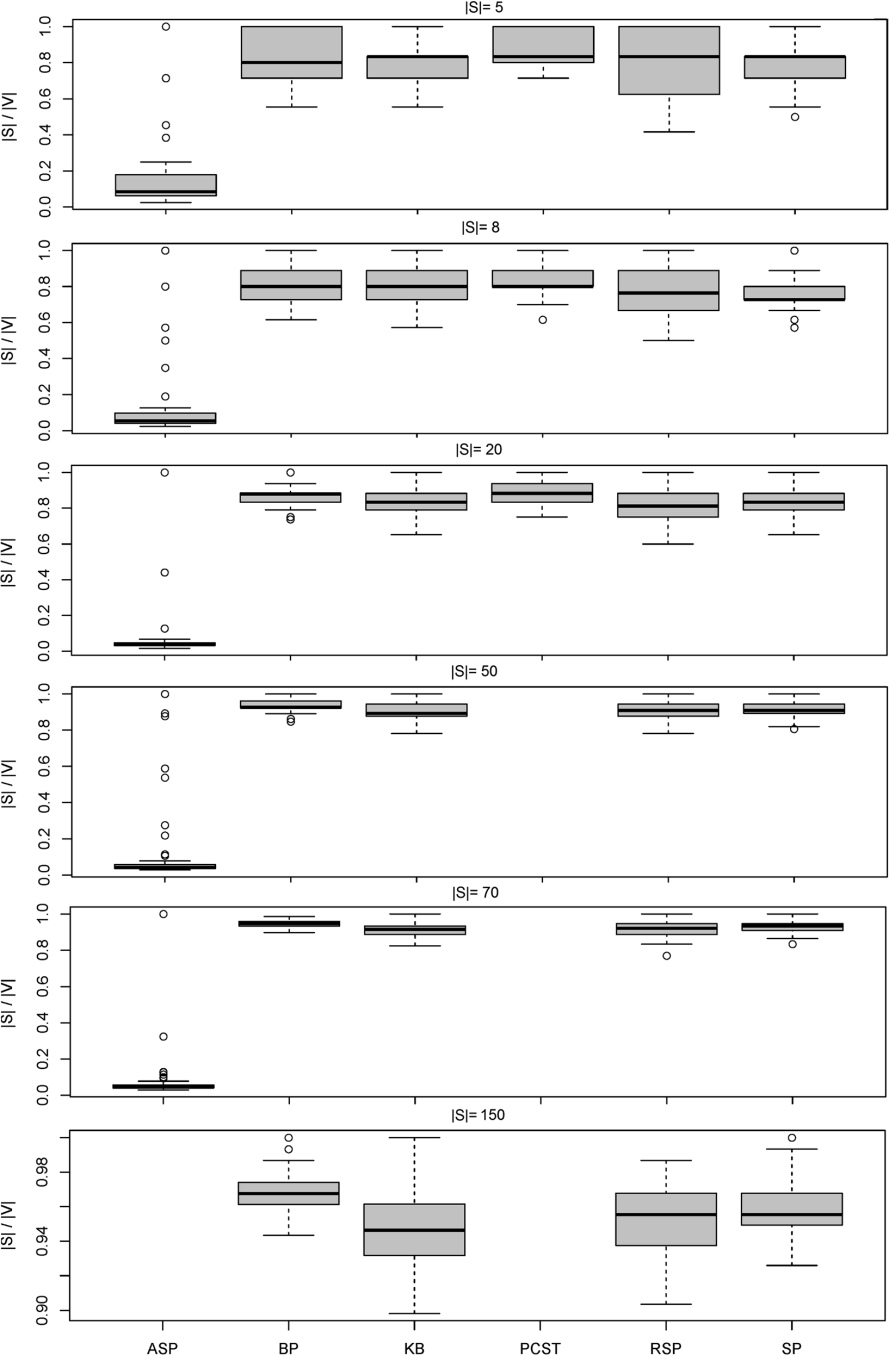
**Terminal frequency (|*S*|/|*V*|) of constructed sub**-**networks with different heuristic Steiner tree algorithms**, **the exact PCST method and the belief propagation approximation (BP).** For |*E*| ≥ 50 the PCST algorithm became impractical slow. The same was true for the ASP algorithm for |*E*| = 150.

**Figure 5:**
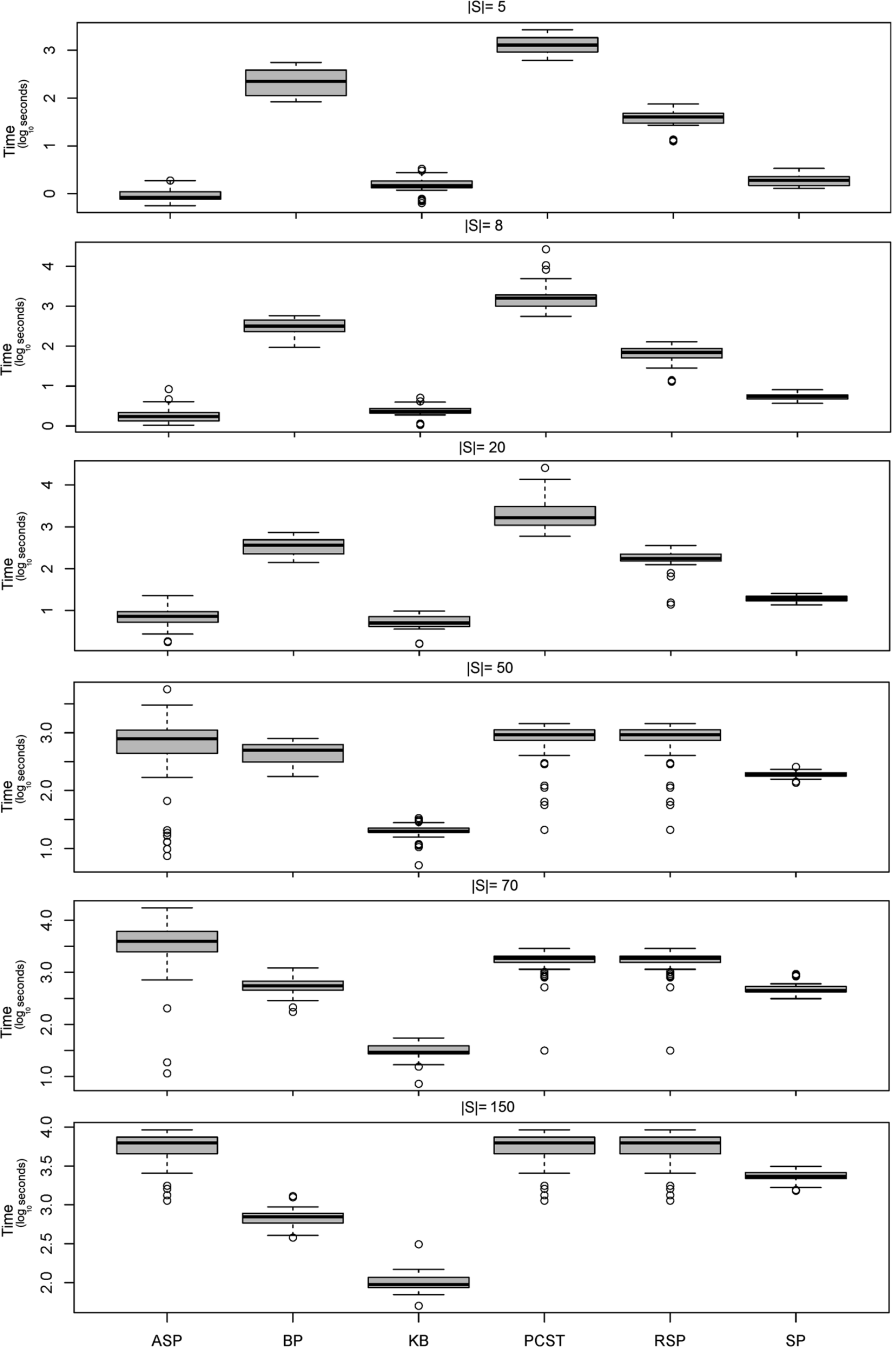
**Computation time (*s*) of different heuristic Steiner algorithms**, **the exact PCST method and the belief propagation approximation (BP) on log_{1 0} scale.** For |*E*| ≥ 50 the PCST algorithm became impractically slow. The same was true for the ASP algorithm for |*E*| = 150.

Our experiment showed that Steiner trees constructed with the SP, KB and RSP heuristics were significantly smaller than those constructed with the ASP method (Figure 3). At the same time the terminal frequency was clearly higher than with the ASP method (Figure 4). Our RSP algorithm performed similar to the SP and KB methods for |*S*| ≤ 50, but had clearly a higher computation time (Figure 5). For |*S*| ≥ 70 RSP yielded significantly larger Steiner trees with lower terminal frequency than the SP algorithm (*p* < 0.05, paired Wilcoxon signed rank test with Holm's correction for multiple testing). For |*S*| ≥ 70 terminals the SP algorithm lead to significantly better solutions than the KB method with respect to the number of edges as well as the edge frequency (*p* < 0.05), otherwise no significant differences could be observed.

The ASP algorithm empirically showed the worst time scaling behavior among all approximate approaches, which can be explained by the high number of edge insertions (see above). For |*S*| = 50 it became so slow that we had to exclude it from our comparison.

Taken together our SP algorithm lead to the highest quality solutions. In our implementation the KB algorithm was faster than SP, but at the cost of a worse quality of solutions for higher number of terminals. In summary the SP algorithm was our overall preferred heuristic due its good compromise between solution quality and computational speed.

### Comparison to an exact and approximate PCST algorithm

We compared the performances of our approximate Steiner tree methods to an exact as well as to an approximate algorithm for the PCST problem. In contrast to the algorithms tested in the last sub-section, the PCST problem deals with weighted graphs, which yields a different algorithmic problem. As an exact PCST algorithm we employed the implementation provided in the R-package BioNet [16], which is based on Integer Linear Programming (ILP) and uses the IBM CPLEX solver. The R-package BioNet contains a wrapper for a C++−code, which compiles the ILP in the format required by CPLEX and returns the solution to the R interface. As an approximate PCST method we used the belief propagation algorithm (BP) by Bailly-Bechet et al. [15], which can be downloaded as a C++ code from their homepage. As a node weighting scheme we used +1 for terminals and −1 for non-terminals in both cases.

**Figure 6:**
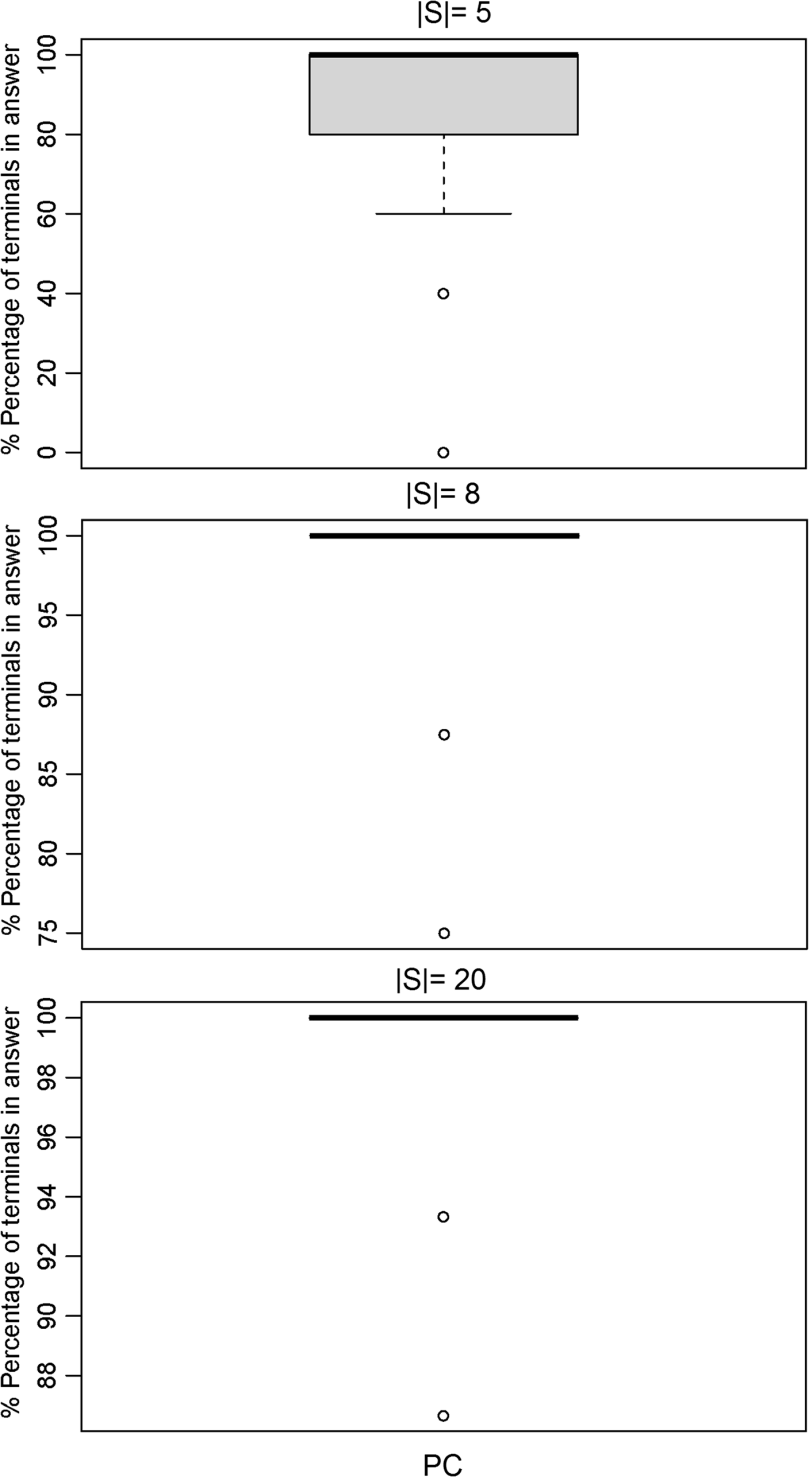
Fraction of terminals included into the solution of the PCST problem (exact method).

Our simulation showed that the exact PCST algorithm (PCST) yielded significantly smaller networks than all approximate Steiner tree algorithms (Figures 3 and 4). However, the computation time was two orders of magnitude higher, although the implementation is mainly in C++ compared to pure R for the approximate ST methods tested here (Figure 5). For |*S*| ≥ 50 the PCST method became so slow that we had to exclude it from further comparisons. Moreover, one has to take into account that the PCST algorithm does not guarantee all terminals to be included into identified sub-networks (Figure 6).

**Figure 7:**
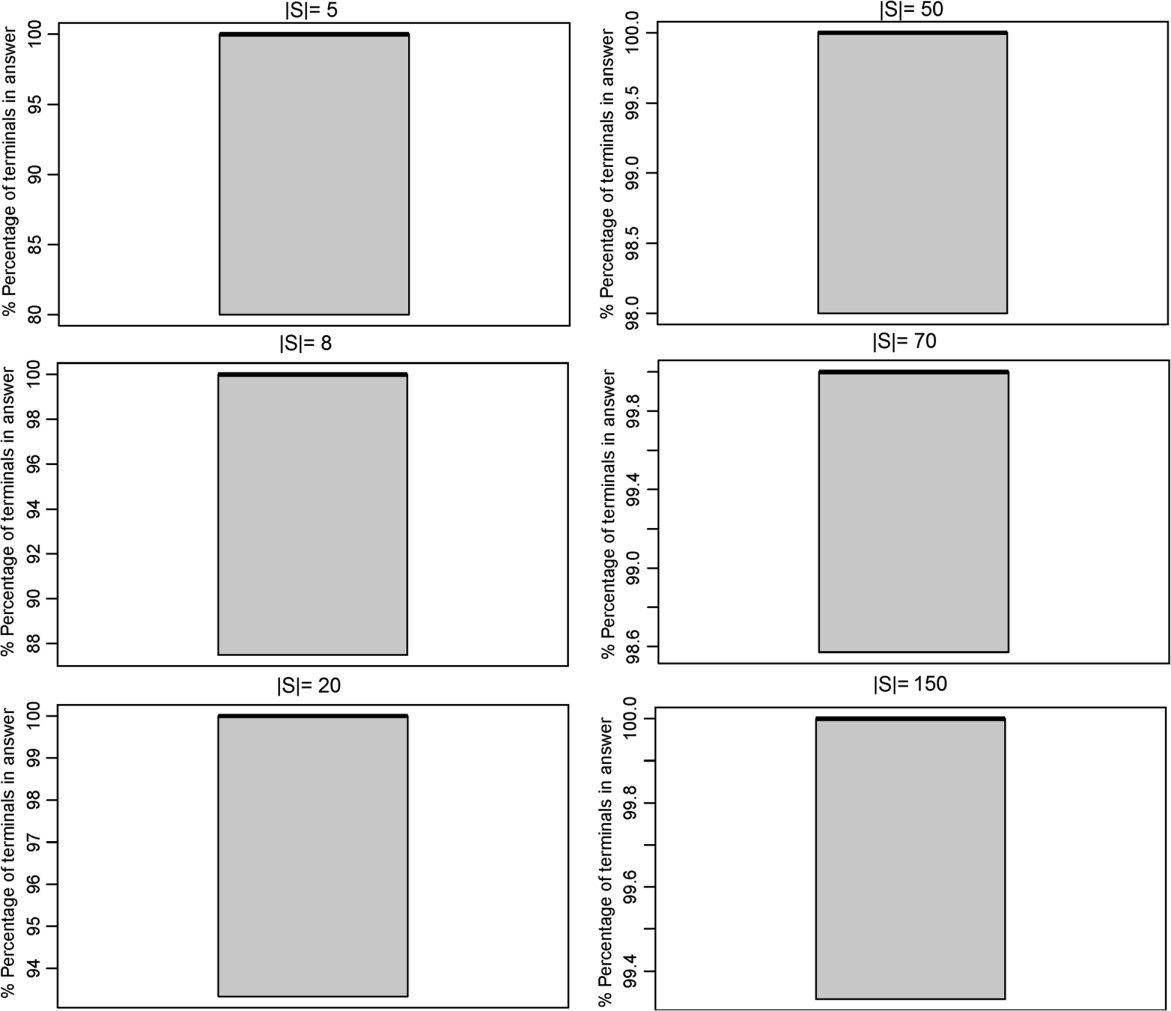
Fraction of terminals included into the solution of the PCST problem (belief propagation).

Not very surprisingly, the belief propagation algorithm (BP) was significantly faster than the exact PCST method, specifically for larger networks (Figure 5). Nonetheless, for |*S*| ≤ 70 *BP* was slower than SP. Again it is to be emphasized here that BP is a highly optimized C++ implementation, whereas the SP method is just implemented in R. Similar to the exact PCST algorithm the BP method yielded significantly smaller sub-networks than all approximate Steiner tree approaches (Figures 3 and 4). However, at the same time the fraction of terminals included into the final solution of the BP method varied between 80 to 100%, depending on the number of terminals (Figure 7).

### Comparison to exact steiner tree algorithm

We next investigated the accuracy of the best of our tested Steiner tree heuristics, namely the shortest paths based algorithm, in comparison to the exact Steiner tree algorithm. Since the exact algorithm becomes infeasible slow for networks with more than ~50 nodes we sampled sub-graphs with at most 30 nodes from our complete network. This was done via our above described random walk. Visited nodes, including all their incident edges and neighbors were joined into one sub-graph. The process was continued until at least 30 nodes were contained in the sub-graph. At the same time terminals were selected as described before. We investigated terminal sets with 5 and 8 proteins. Figure 7 shows the size distributions of sampled sub-graphs for both cases.

**Figure 8:**
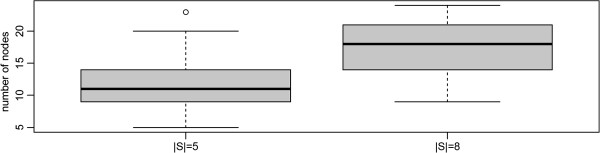
Size of sampled sub-networks (number of nodes).

**Figure 9:**
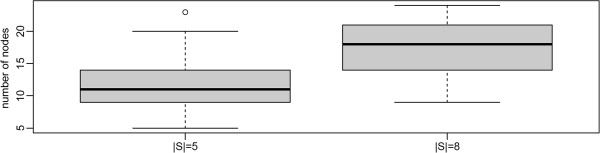
**Comparison of the SP approximation to the exact solution: network size (|*E*|).**

**Figure 10:**
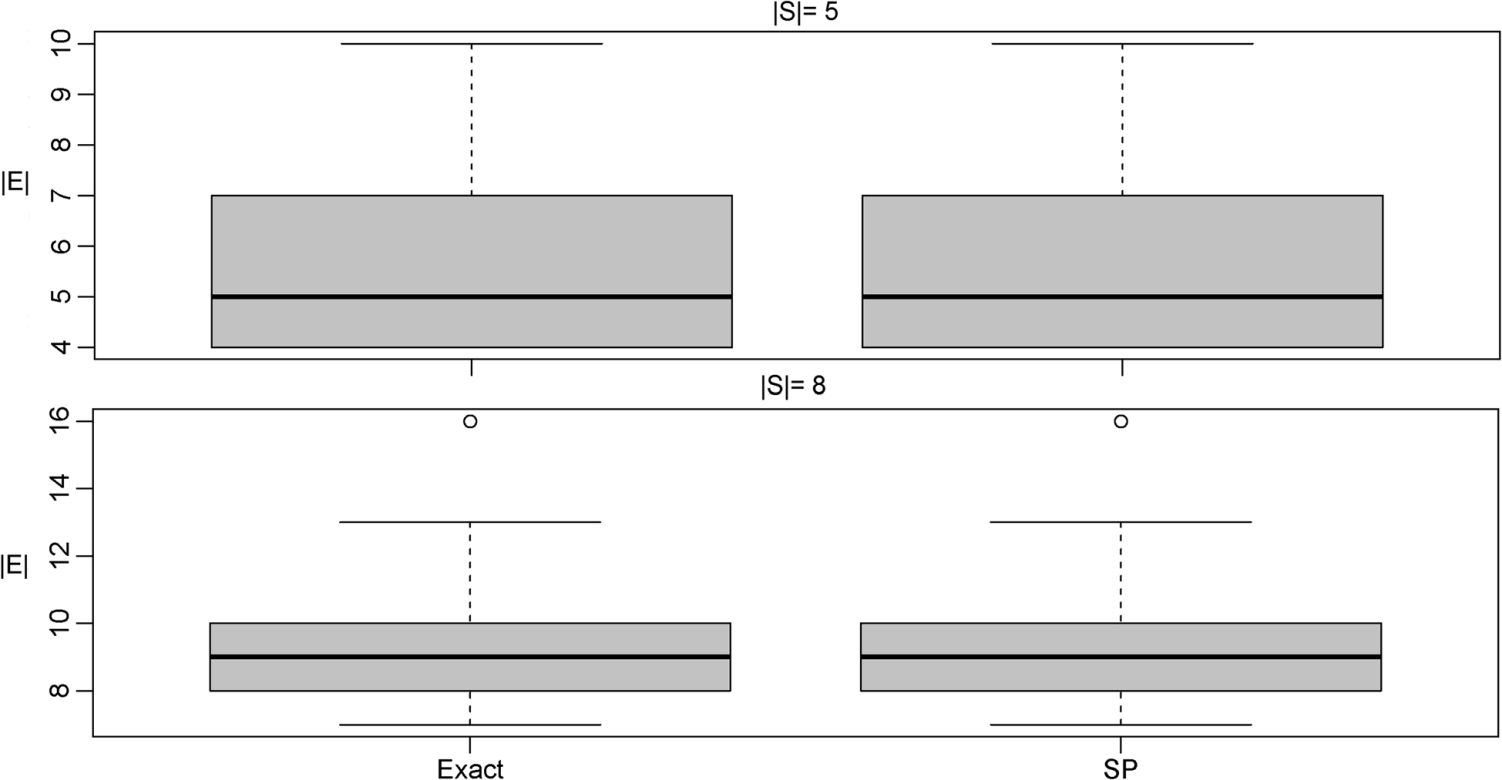
**Comparison of the SP approximation to the exact solution: terminal frequency (|*S*| = |*V*|).**

**Figure 11:**
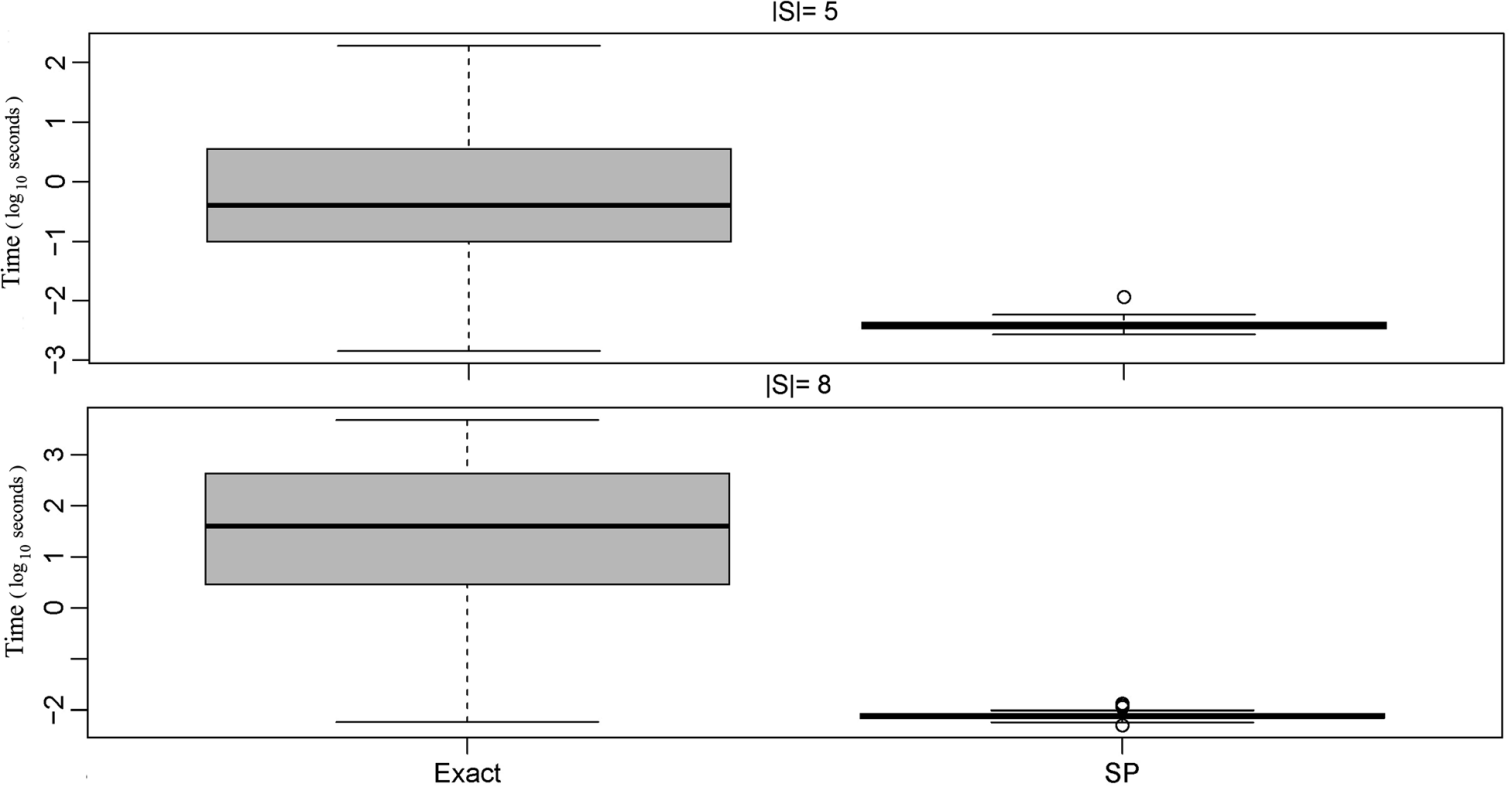
Comparison of the SP approximation to the exact solution: computation time.

No significant difference in terms of terminal frequency and Steiner tree sizes could be observed between the SP and the exact algorithm, which was ~4 orders of magnitude faster (Figures 8, 9, 10, 11).

### Merged steiner trees

**Figure 12:**
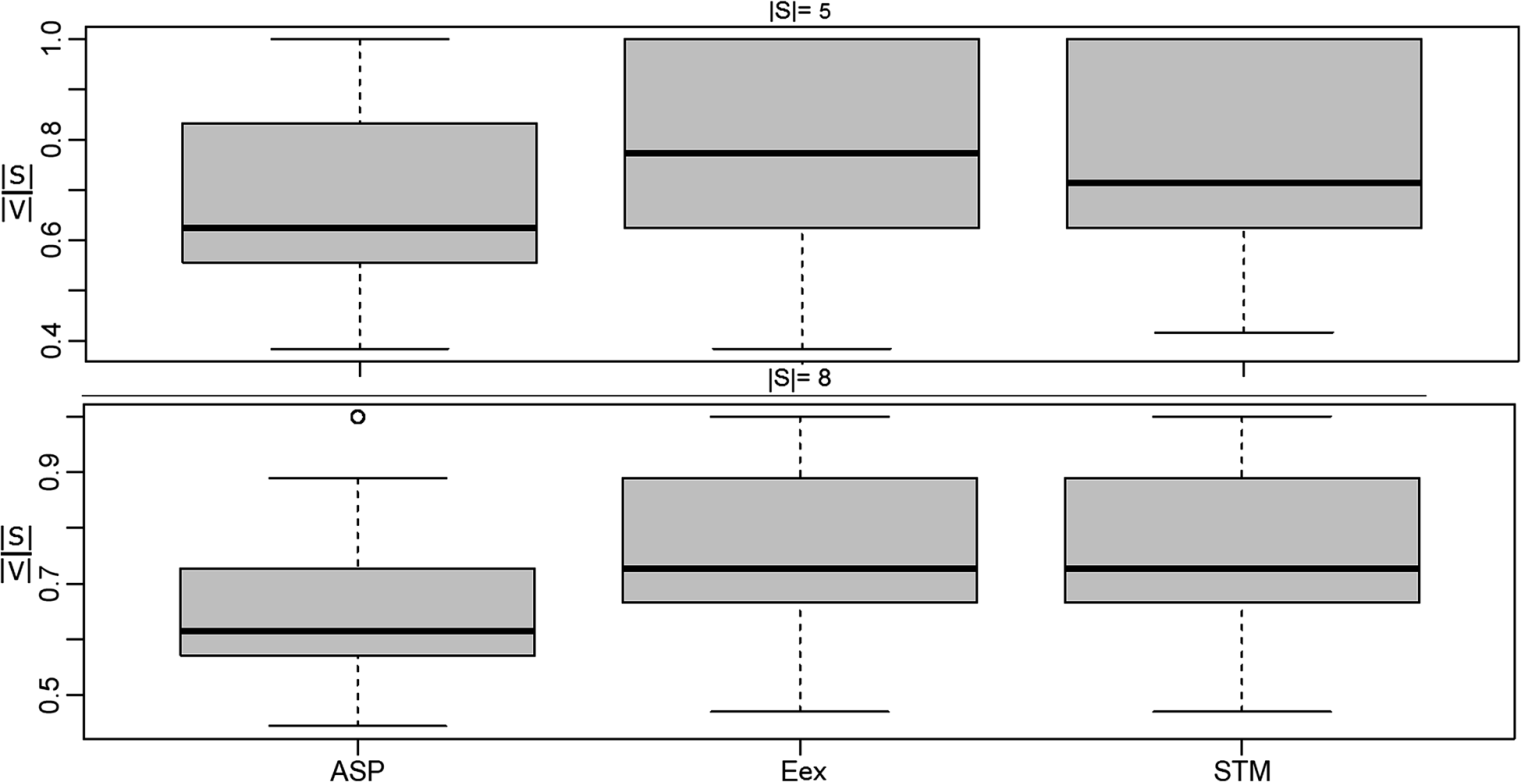
**Merged Steiner Trees: Comparison of the ASP and STM approximations to the exact solution in terms of network size (|*E*|).**

**Figure 13:**
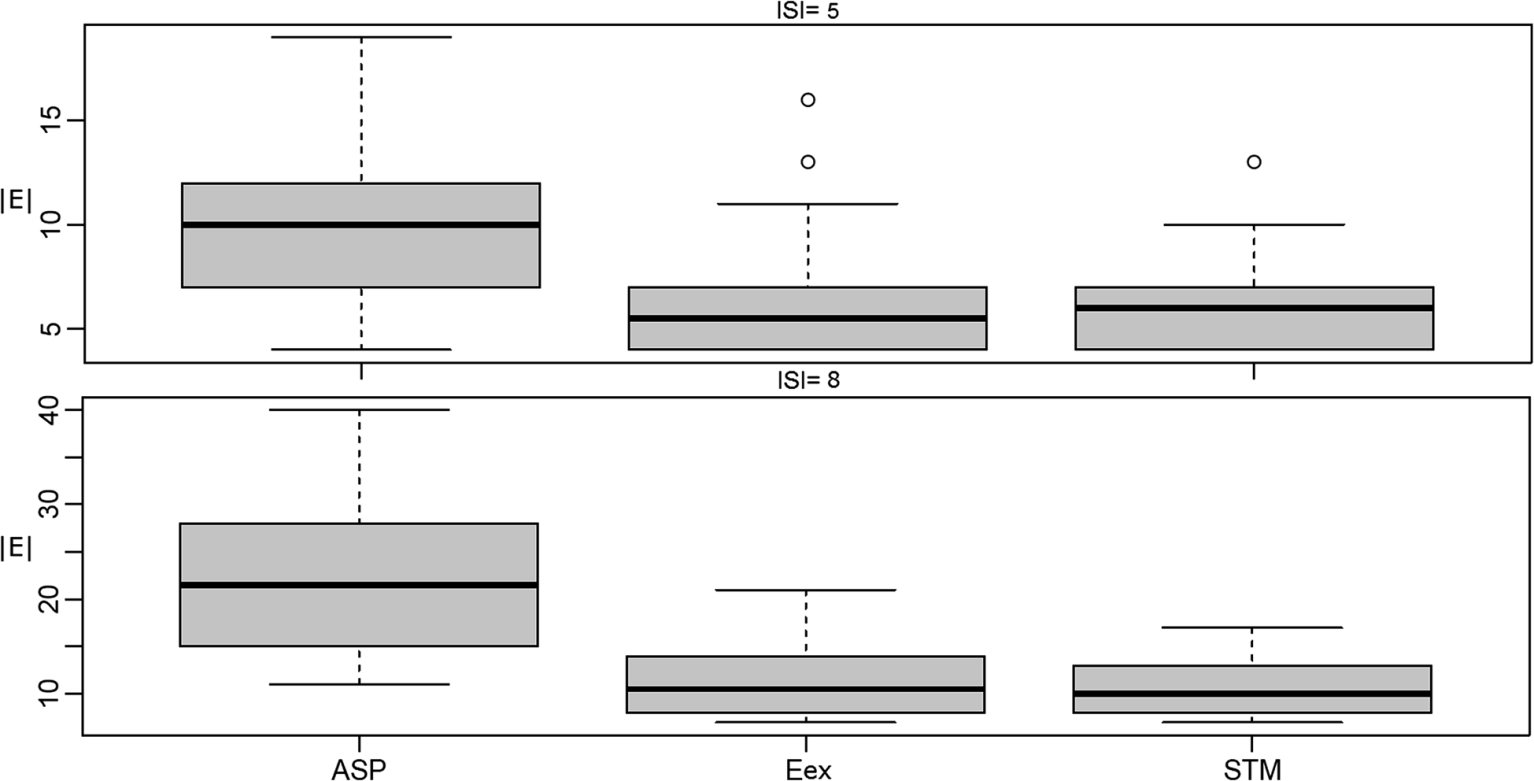
**Merged Steiner Trees: Comparison of the ASP and STM approximations to the exact solution in terms of terminal frequency (|*S*| = |*V*|).**

**Figure 14:**
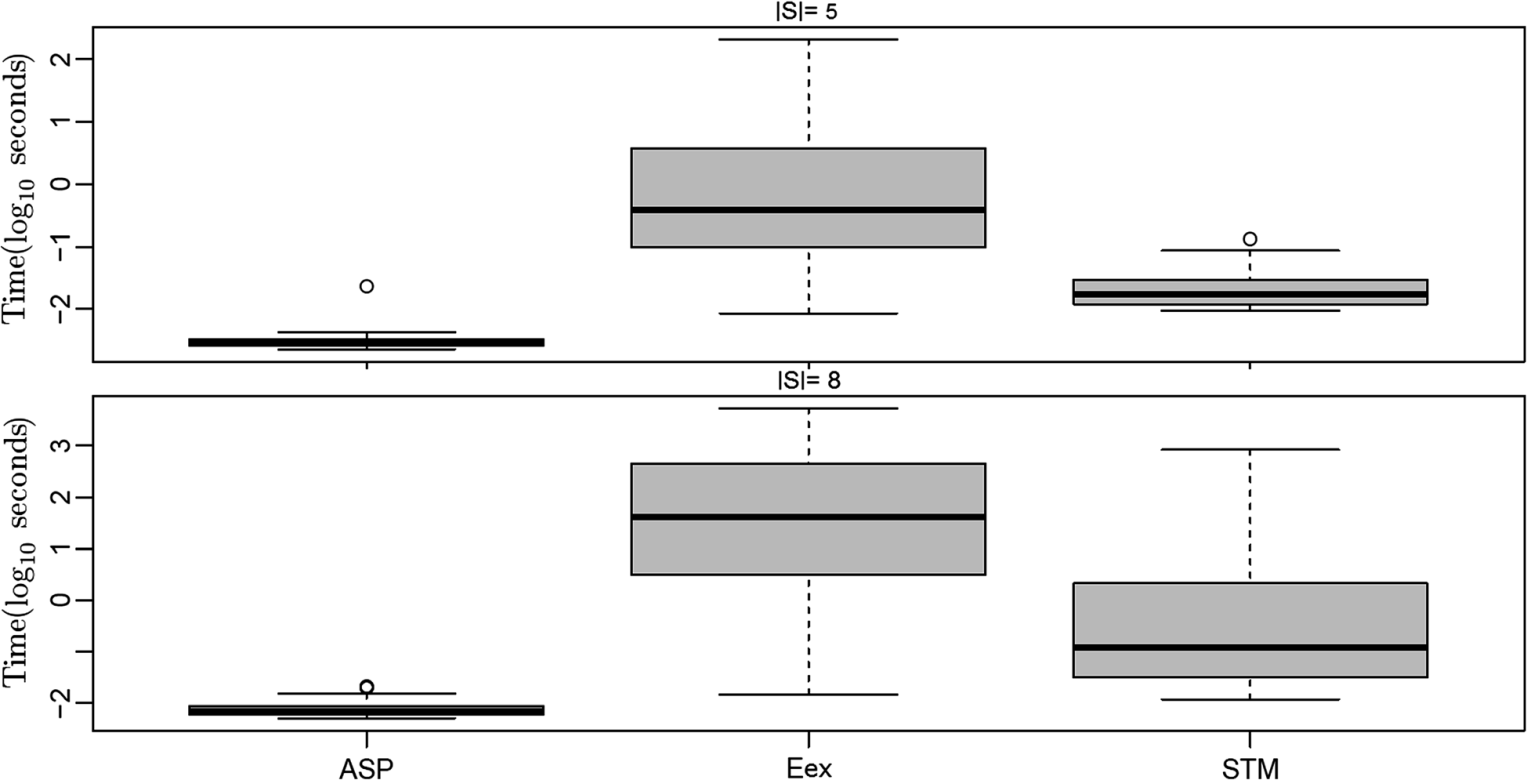
Merged Steiner Trees: Comparison of the ASP and STM approximations to the exact solution in terms of computation time.

Finally we compared our modified shortest paths based approximation producing a sub-graph of merged Steiner trees (STM) against the exact approach. The same simulation setup as described in the last section was employed. Similar to before this comparison revealed no significant difference between STM and the exact algorithm, while at the same time STM was almost 3 orders of magnitude faster (Figures 12, 13, 14).

Compared to the ASP algorithm, which joins all shortest paths between terminals, the STM method produced significantly smaller sub-graphs with higher terminal frequency (Figures 11 and 12).

### Dependency of simulation results on terminal selection probability

We investigated, in how far our shown simulation results were dependent on the terminal selection probability, which affects, how close terminals are to each other. The larger the smaller the distance between terminals. We repeated the whole set of simulations presented here with *θ* = 0.2 and *θ* = 0.8 (see Additional file 1). In both cases SP was statistically significant outperforming the KB algorithm for |*S*| ≥ 50. Only in one case (|*S*| = 8 and *θ* = 0.2) the SP algorithm was leading to significantly worse solutions than the exact algorithm, otherwise no major differences could be detected. Moreover, our STM method was always leading to solutions at comparable quality to the exact algorithm.

### Male pattern baldness

In a recent GWAS study *HDAC*9 was found to be associated with male pattern baldness [19]. Mining known protein-protein interactions via PathwayCommons [4] and with the help of the commercial software MetaCore^TM^ the authors established an indirect connection of *HDAC*9 to the androgen receptor (*AR*). We used both, our approximate STM as well as the exact algorithm to construct a sub-graph of merged Steiner trees between *HDAC*9 and the *AR* (Figure 2). Both algorithms produced exactly the same result, which highlights multiple indirect interactions on protein level between both molecules.

### 70-Gene prognostic breast cancer signature

**Figure 15:**
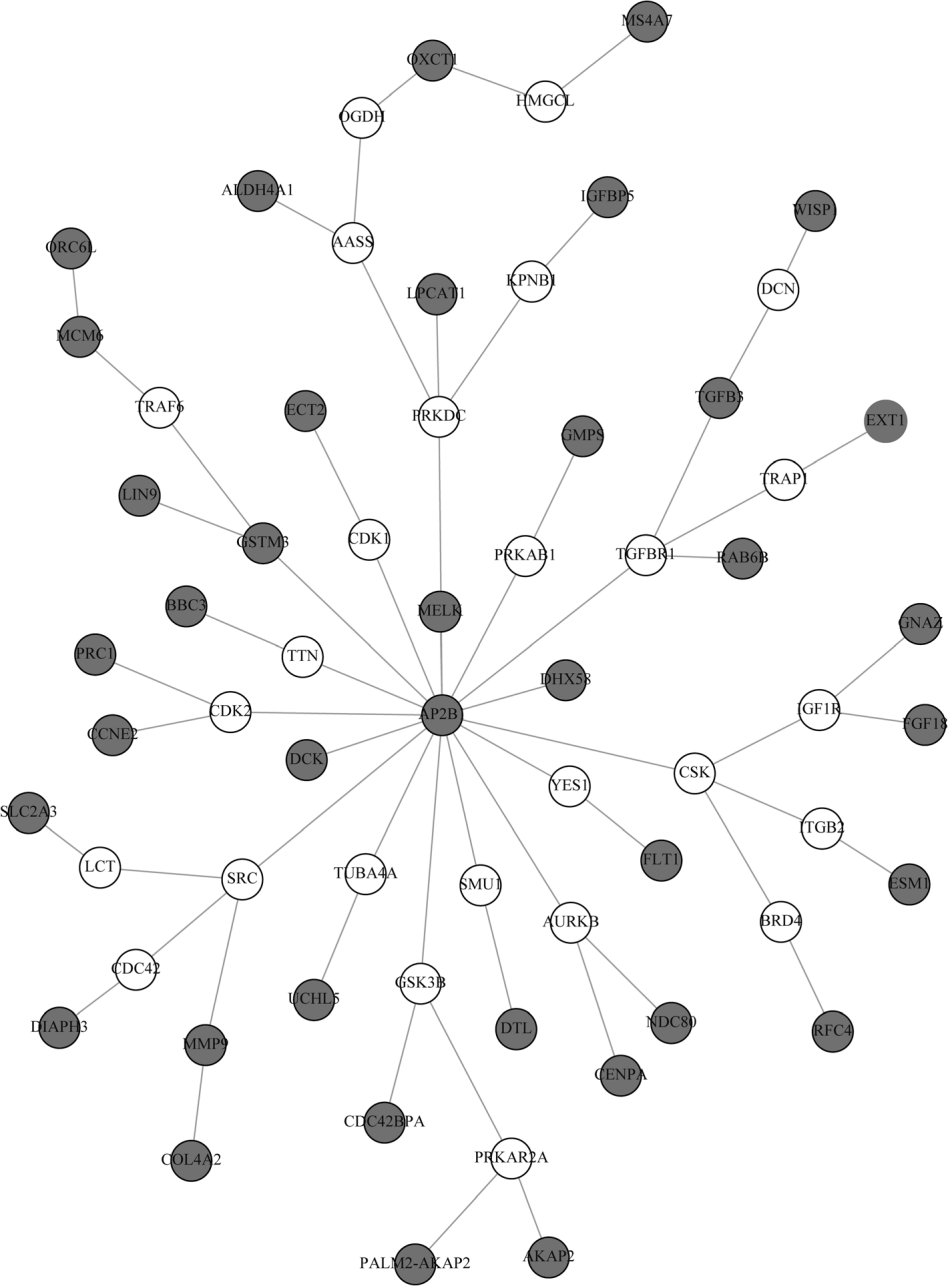
Sub-network reconstructed by the SP algorithm for the prognostic 70 gene breast cancer signature (gray = terminal nodes).

The well known 70 gene signature for bad vs. good breast cancer prognosis established by van't Veer et al. [20] was downloaded from GeneSigDB [40]. 38 genes from the original signature could be mapped to our network. Application of the SP algorithm yielded a sub-network with 64 proteins (Figure 15), compared to 65 proteins with the KB, 68 proteins with the RSP and 61 proteins with the BP algorithm (2 Figures in Additional file 1). The network obtained by the exact PCST algorithm had only 40 nodes, but contained just 28 out of 38 terminals (see Additional file 1). A Gene Ontology (GO) [41] analysis using a hyper-geometric test conditioned on the GO structure [42] was applied to the network retrieved via the SP algorithm and revealed a significant enrichment of cell cycle genes (*p* < 0.05).

### 286-Gene invasive breast cancer signature

**Figure 16:**
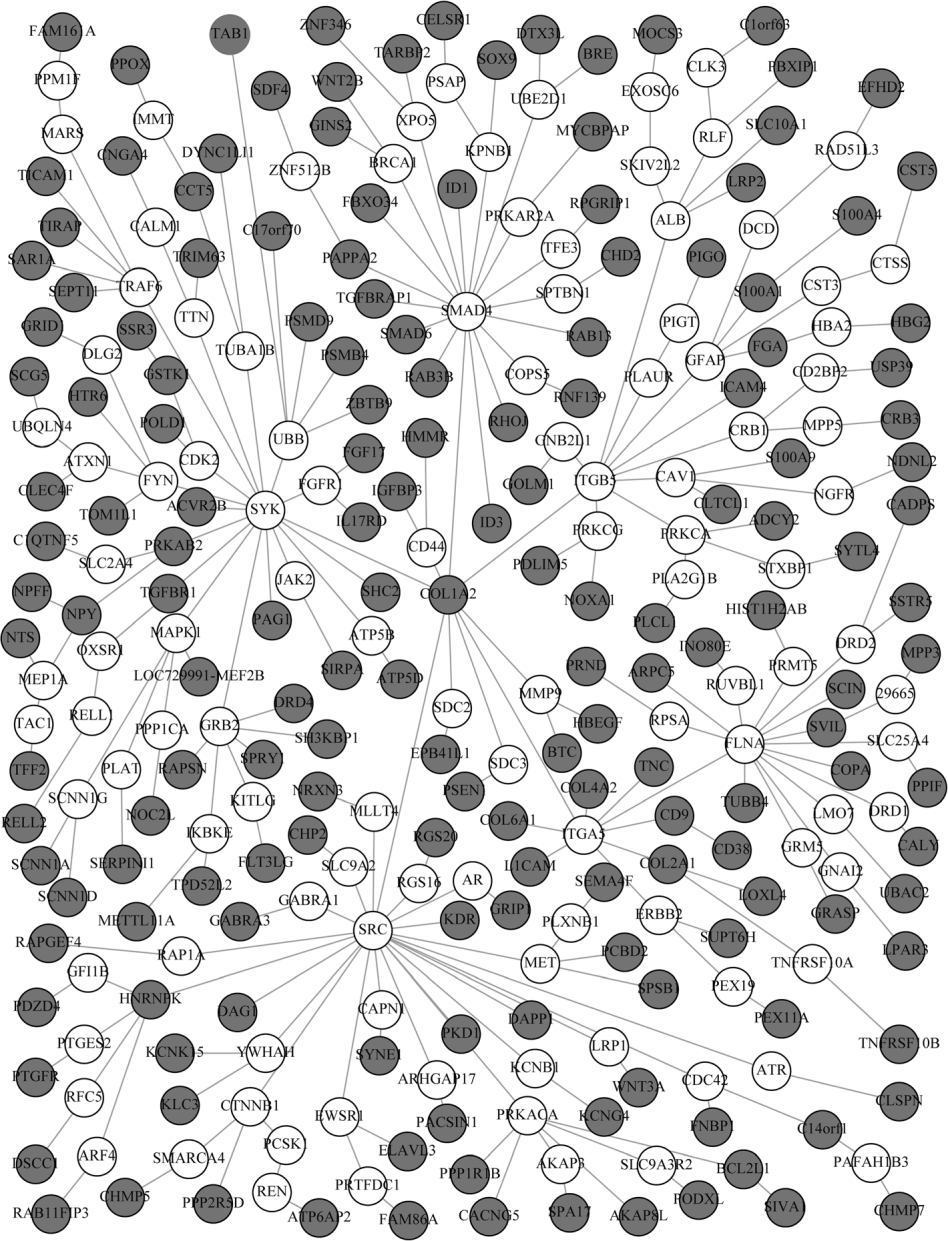
Sub-network reconstructed by the SP algorithm for the 286 gene invasive breast cancer signature (gray = terminal nodes).

As a third show case we investigated the 286 gene signature published by Wang et al. [21], which correlates with poor prognosis in invasive breast cancer. Again the signature was downloaded from GeneSigDB. 170 genes from the original signature could be mapped to our network. Application of the SP algorithm yielded a sub-network with 282 proteins (Figure 16), compared to 283 with the KB, 289 proteins with the RSP and 255 proteins with the BP algorithm (see Additional file 1). The exact PCST algorithm was stopped after one week of computation without result. The SP network was found to be enriched for cell localization, positive regulation of transferase activity, regulation of hormone levels, insulin secretion and cellular component morphogenesis (all *p* < 0.05). Transferase activity has been shown to be related to breast cancer [43] as well as the level of insulin secretion [44].

## Discussion and conclusion

Identification of an optimal sub-network connecting a list of seed proteins provides valuable insights for the interpretation of experimental data, setting up system biological models, planning novel experiments as well as generating prior knowledge for advanced statistical learning methods. Steiner tree algorithms provide a theoretically well founded approach to address this task. Whereas in the computer science literature Steiner tree methods have been theoretically well studied, their practical application to large molecular interaction networks has not been investigated systematically. This is specifically relevant in the light of the NP completeness of the Steiner tree problem, which makes exact algorithms in many cases impractical. Here we tried to fill this gap via an empirical study comparing different approximate Steiner tree algorithms with each other as well as to an exact algorithm. We specifically focused on the sub-network identification problem in *unweighted* graphs. Our systematic simulations revealed that a modified version of the shortest paths based heuristic by Takahashi and Matsuyama yielded satisfactory solutions at a reasonable computational effort, which was several orders of magnitude below that of an exact solution. Compared to using the belief propagation method by Bailly-Bechet al. or other algorithms solving the PCST problem this has the advantage that the inclusion of all terminal nodes in the solution can be guaranteed. There is no need to define a node weighting scheme, which in the absence of any clear information always becomes rather arbitrary. Of course, this situation changes, if experimental data allows the specification of a weighted graph in an obvious manner. In such a case application of PCST methods becomes much more natural and has proven to be useful in several papers [10,15]. Nonetheless, one should even then still be aware of the fact that the solution does not necessarily contain all terminal nodes.

We demonstrated the usefulness of approximate Steiner tree methods in two breast cancer studies. Application of the shortest path algorithm here lead to compact and at least in case of the 286 gene signature also to clearly biologically interpretable networks.

In contrast to most other authors we also payed attention to the fact that Steiner trees of a given size are not necessarily unique. This was demonstrated via an example from a GWAS study regarding male pattern baldness here. We proposed an own heuristic algorithm, which was a further modification of the shortest path based heuristic, for this purpose. Our method was found to be highly accurate and significantly faster than an exact approach. It should be noted, however, that our STM algorithm is typically an order of magnitude slower than the SP method, which only finds one Steiner tree. It is thus only recommended for small seed lists.

We have implemented all of our methods in an R-package SteinerTree, which is freely available from the CRAN repository (http://www.r-project.org) and as a supplement to this paper (Additional file 2).

## Endnotes

^a^http://www.pathwaycommons.org/pc/sif_interaction_rules.do.

## Competing interests

Both authors declare that they have no competing interests.

## Authors’ contributions

The project was initiated and guided by HF. AS invented the RSP algorithm and performed all programming work. Both authors wrote the manuscript together.

## Supplementary Material

Additional file 1 Supplement: Steiner tree Methods for Optimal Sub-Network Identification: an Empirical Study. Click here for file

Additional file 2 R-Package SteinerNet. Click here for file
