# Supplementary material for: Steiner tree methods for optimal sub-network identification: an empirical study
Source: BMC Bioinformatics. 2013 Apr 30;14:144. doi: 10.1186/1471-2105-14-144 (PMC3674966; doi:10.1186/1471-2105-14-144)
Supplement: Additional file 1 — Supplement: Steiner tree Methods for Optimal Sub-Network Identification: an Empirical Study. [file 1471-2105-14-144-S1.pdf]

Supplement: Steiner tree Methods for Optimal Sub-Network Identification: an Empirical Study

Afshin Sadeghi, Holger Fröhlich

March 18, 2013

1 Simulation Results with Terminal Selection Probability 0.2

Figures 1 and 2 depict the size and the terminal frequency of constructed Steiner trees for different heuristic algorithms, if the terminal selection probability is $\theta = 0.2$. Besides the differences of ASP to all other methods we found SP to significantly outperform KB with respect to tree size as well as terminal frequency for $|S| \geq 50$ ($p < 5\%$, paired Wilcoxon signed rank test with Holm's correction for multiple testing). Moreover, SP was always superior to RSP ($p < 5\%$), but lead to significantly larger networks than PCST ($p < 1\%$). The fraction of terminals included into the PCST solution was only 80% for 5 and 90% for 8 terminals. With 20 terminals the median was almost 100% with an IQR going down to 80%.

For $|S| = 8$ terminals the difference of the SP algorithm to the exact algorithm with respect to tree size and terminal frequency was significant (Figures 3, 4). No significant difference between STM and the exact algorithm could be observed (Figures 5, 6).

Figure 7 depicts the fraction of terminals, which was included into the solution by the exact price collecting Steiner tree (PCST) algorithm. Figure 8 depicts the same for the belief propagation (BP) algorithm.

2 Simulation Results with Terminal Selection Probability 0.8

Figures 9 and 10 depict the size and the terminal frequency of constructed Steiner trees for different heuristic algorithms, if the terminal selection probability is $\theta = 0.8$ We found SP to significantly outperform KB with respect to tree size as well as terminal frequency for $|S| \geq 50$ ($p < 1\%$, paired Wilcoxon signed rank test with Holm's correction for multiple testing). For $|S| = 50$ and $|S| = 70$ RSP was significantly better than SP. The PCST algorithm yielded

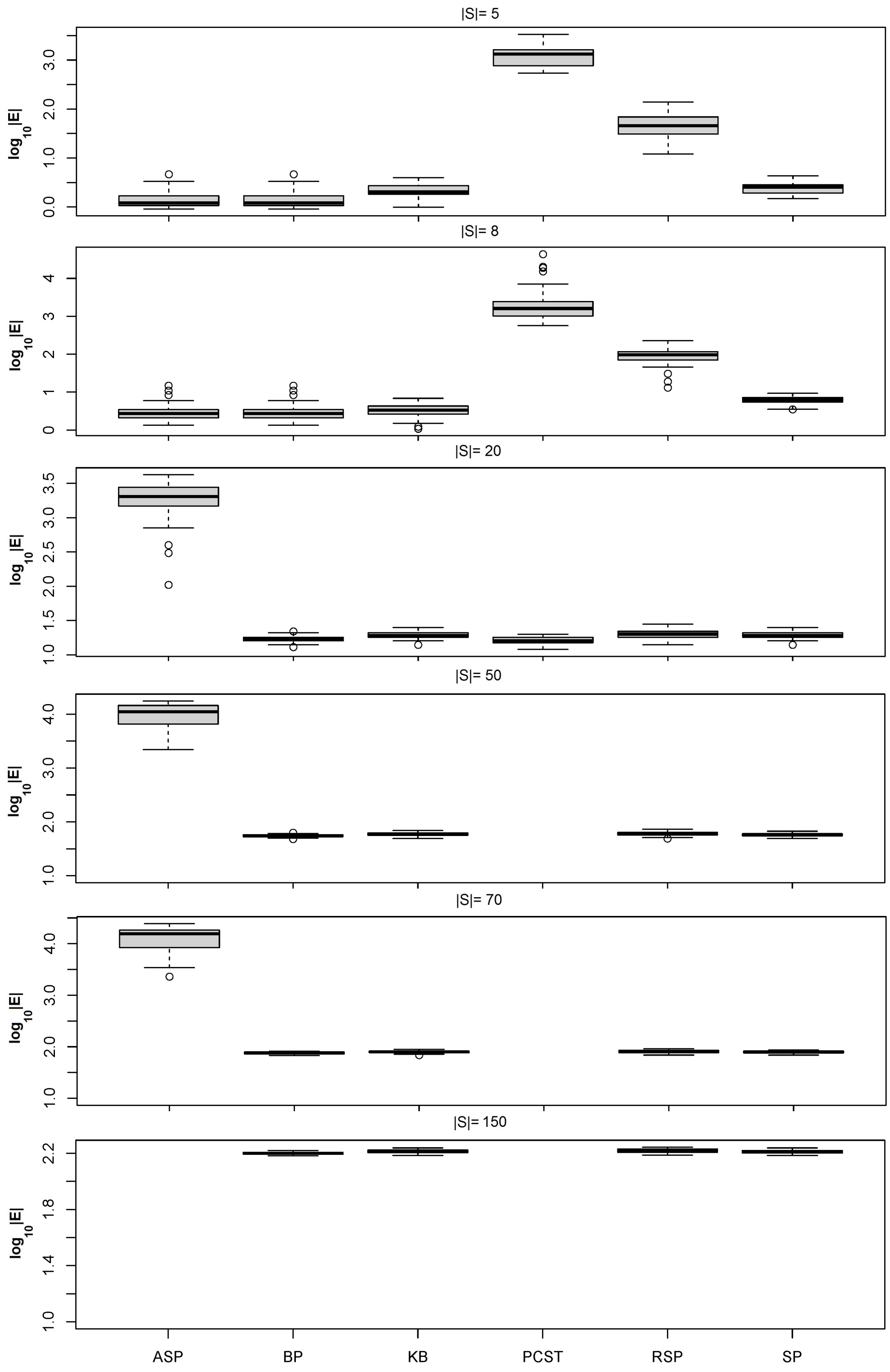

Figure 1: Size ($|E|$) of constructed sub-networks with different heuristic ST algorithms, the exact PCST method and the belief propagation approximation (BP) for $\theta = 0.2$. For $|E| \geq 50$ the PCST method became computationally infeasible for our simulations. The same was true for ASP for $|E| = 150$.

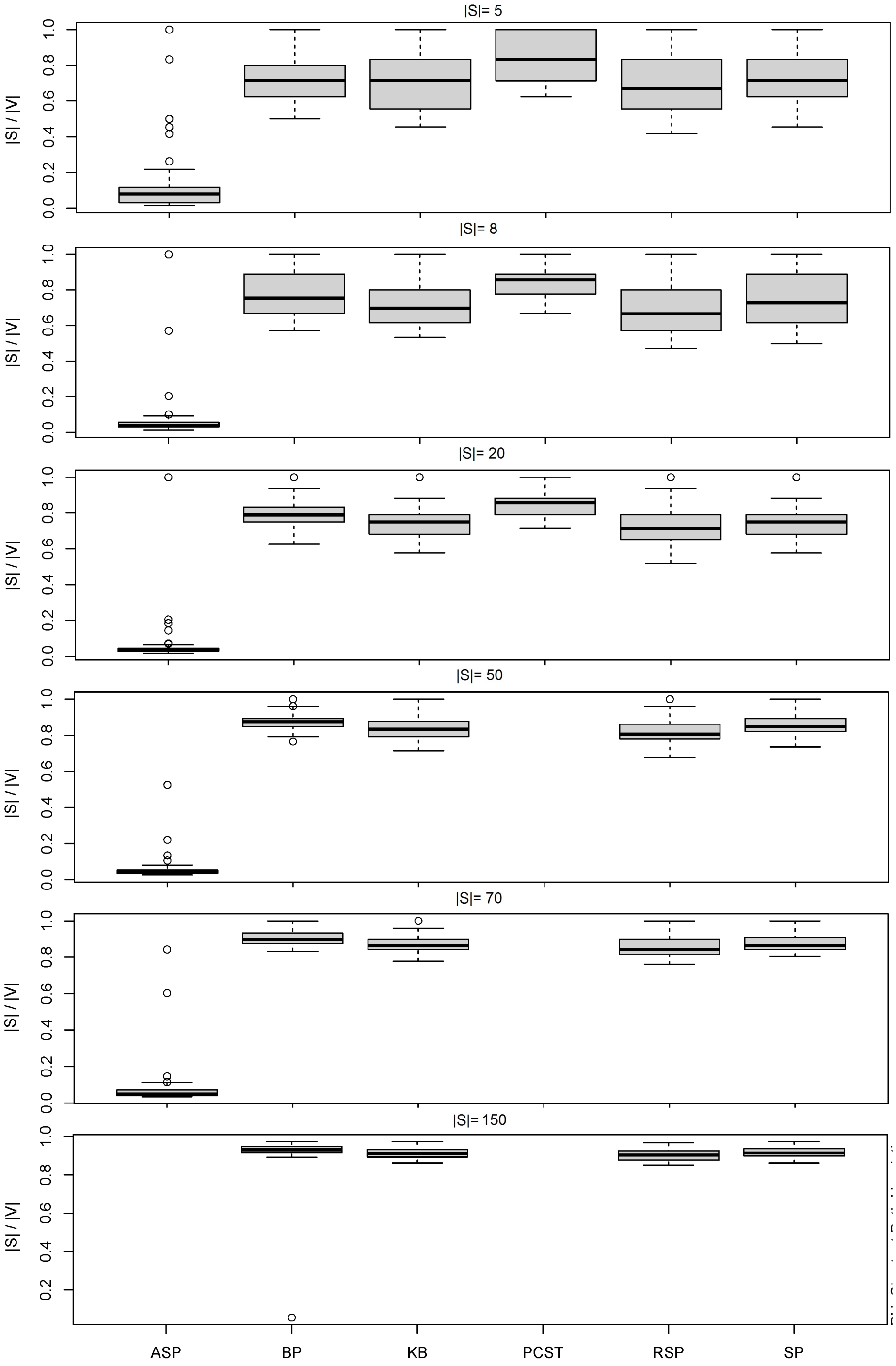

Figure 2: Terminal frequency ($|S|/|V|$) of constructed sub-networks with different heuristic ST algorithms, the exact PCST method and the belief propagation approximation (BP) for $\theta = 0.2$. For $|E| \geq 50$ the PCST method became computationally infeasible for our simulations. The same was true for ASP for $|E| = 150$.

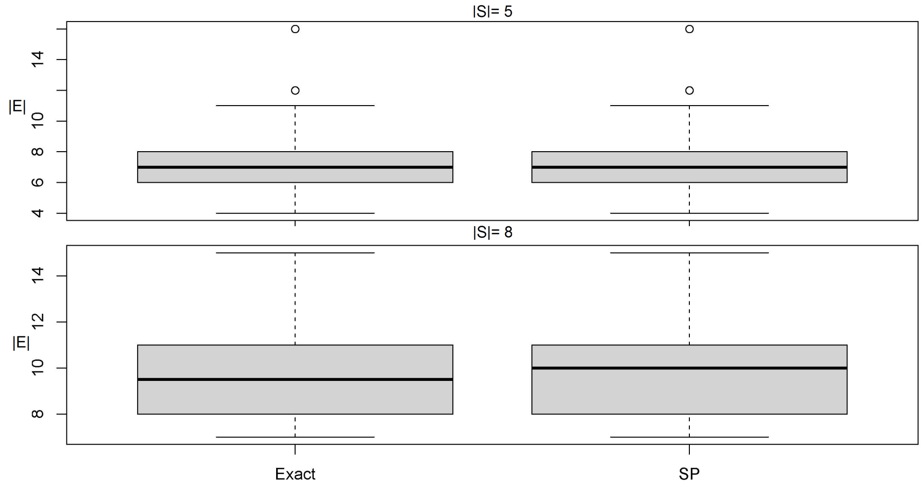

Figure 3: Comparison of the SP approximation to the exact solution: network size ($|E|$) for $\theta = 0.2$.

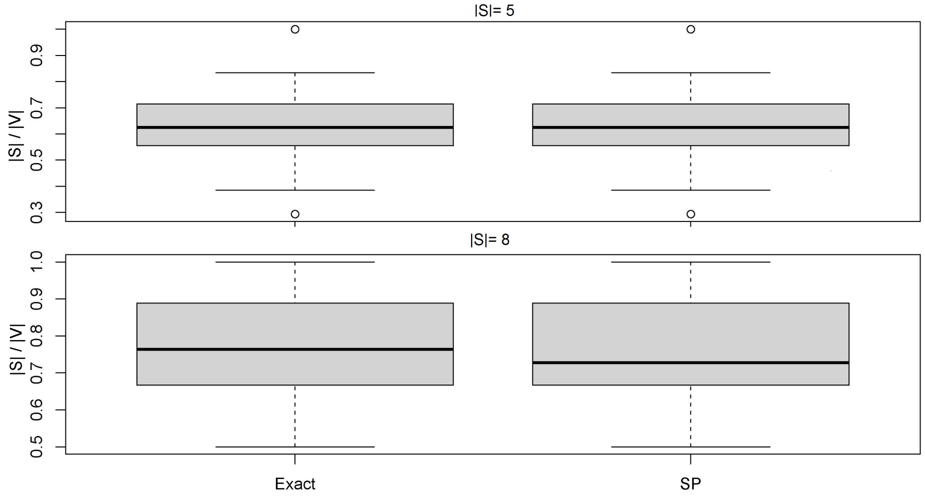

Figure 4: Comparison of the SP approximation to the exact solution: terminal frequency ($|S|/|V|$) for $\theta = 0.2$.

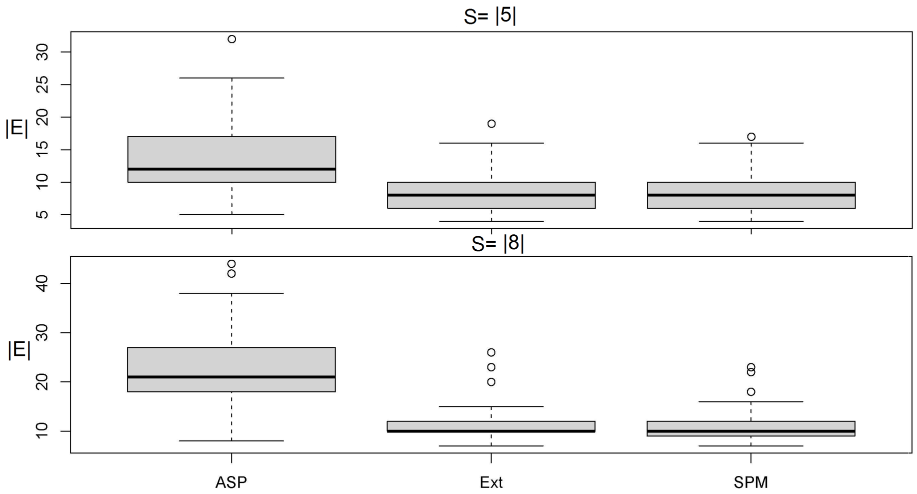

Figure 5: Merged Steiner Trees: Comparison of the ASP and STM approximations to the exact solution in terms of network size ($|E|$) for $\theta = 0.2$.

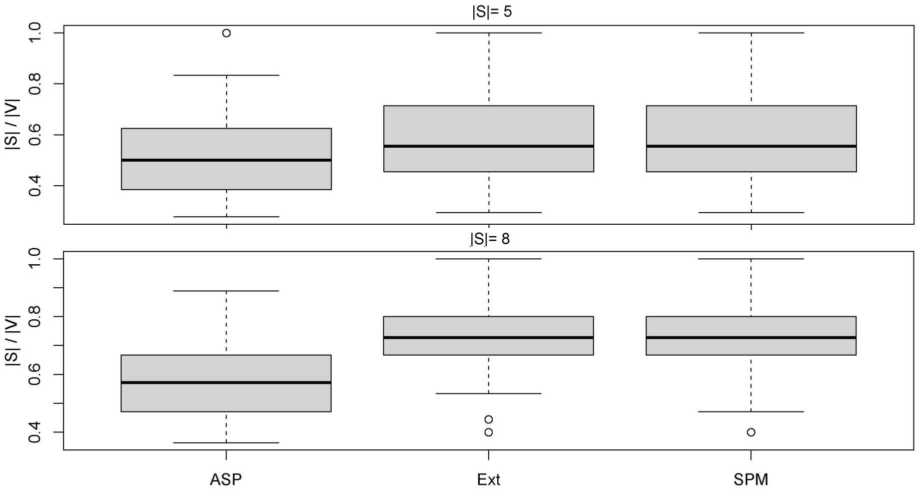

Figure 6: Merged Steiner Trees: Comparison of the ASP and STM approximations to the exact solution in terms of network size ($|E|$) for $\theta = 0.2$.

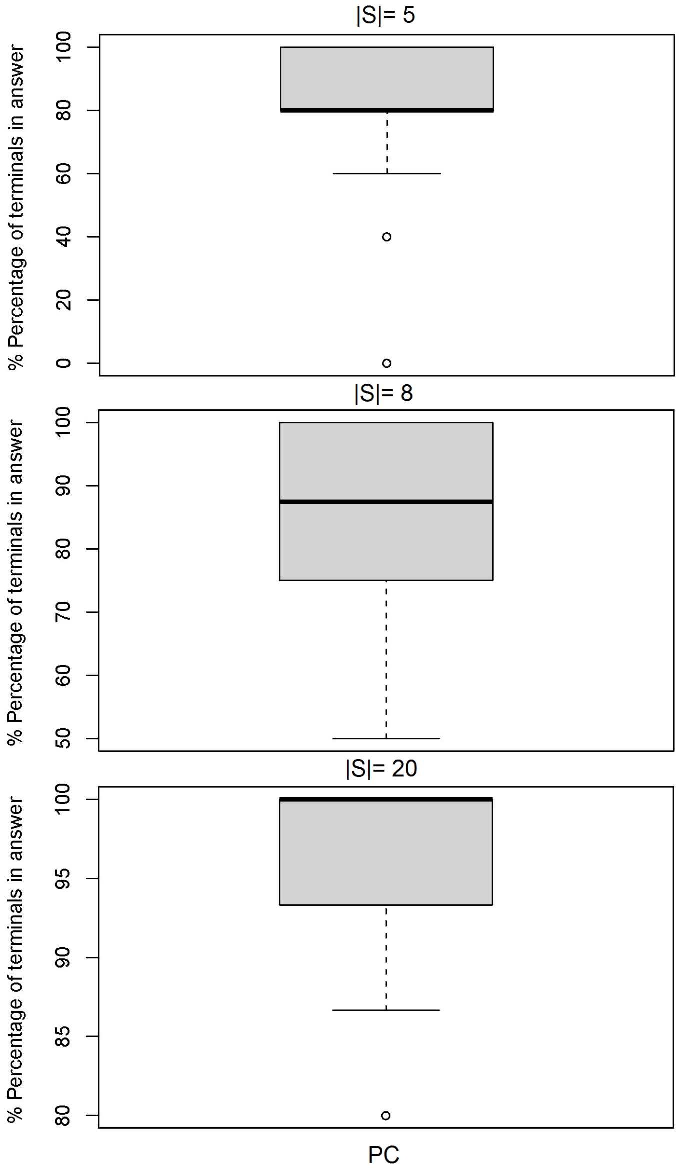

Figure 7: Fraction of terminals recovered by the exact Price Collecting Steiner Tree algorithm for $\theta = 0.2$.

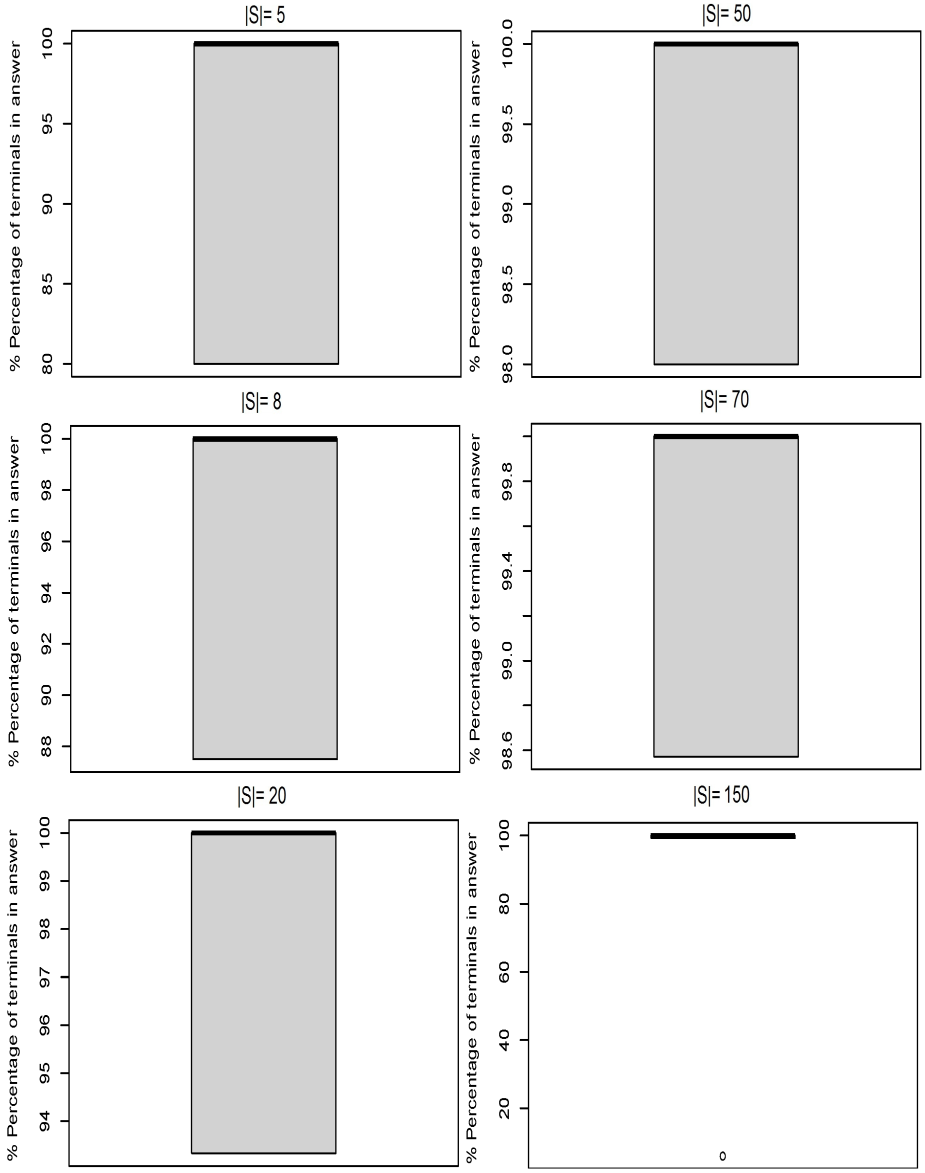

Figure 8: Fraction of terminals recovered by the belief propagation algorithm for $\theta = 0.2$.

significantly smaller networks than the SP algorithm for $|S| = 8$ and $|S| = 20$. The fraction of terminals included into the network in most cases was 100%.

No significant difference between SP and the exact algorithm could be observed (Figures 11, 12). The same was true for STM and the exact algorithm (Figures 13, 14).

Figure 7 depicts the fraction of terminals, which was included into the solution by the exact price collecting Steiner tree (PCST) algorithm. Figure 16 depicts the same for the belief propagation (BP) algorithm.

3 70-Gene Prognostic Breast Cancer Signature

Figures 17 - 20 contain the sub-networks reconstructed via the KB, RSP and PCST algorithm for the 70-gene prognostic gene signature.

4 286-Gene Invasive Breast Cancer Signature

Figures 21 - 23 show the results for the KB and RSP algorithm for the 286-gene invasive breast cancer signature. The calculation of the PCST algorithm was stopped after one weeks without result.

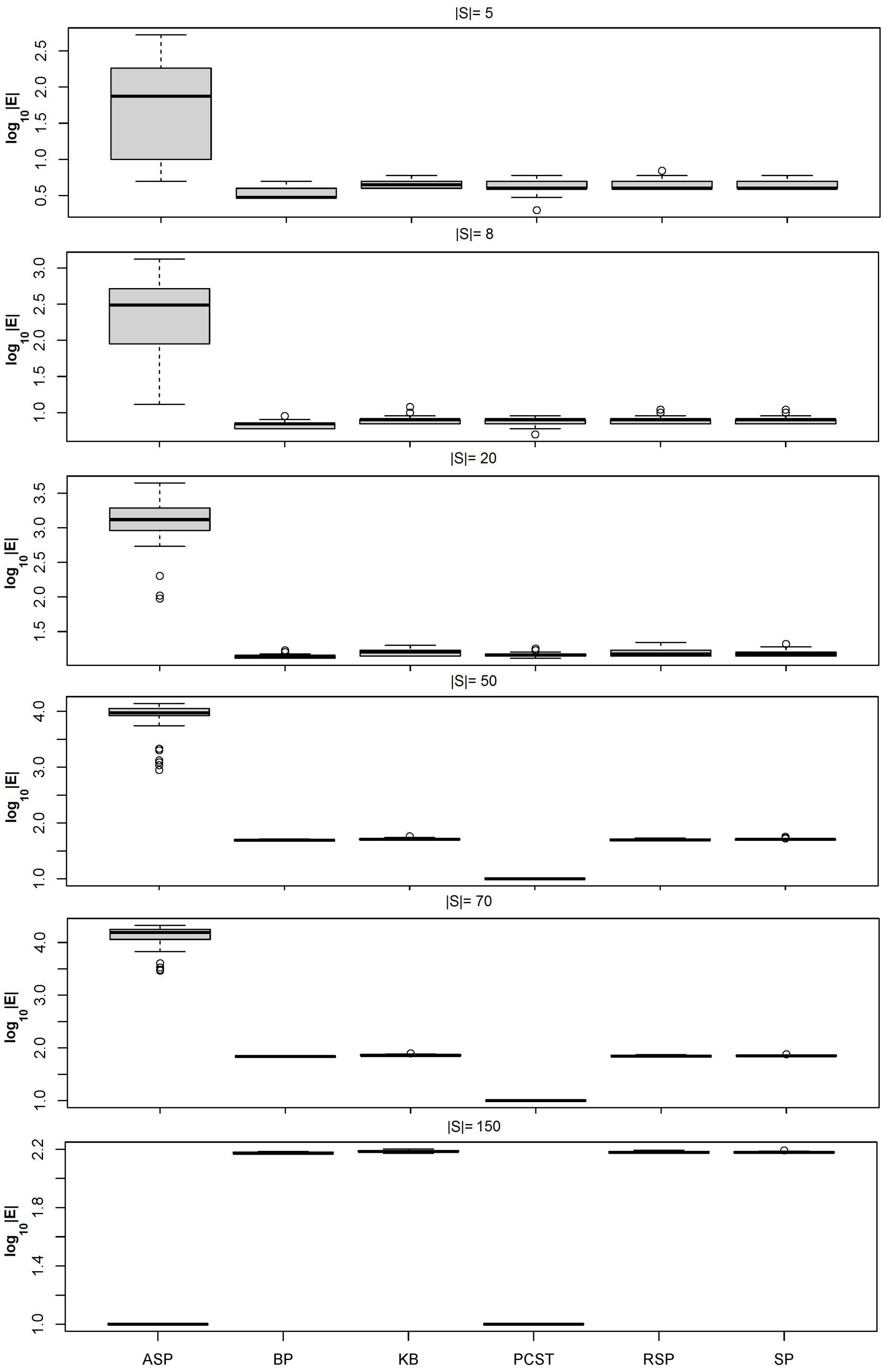

Figure 9: Size ($|E|$) of constructed sub-networks with different heuristic algorithms, the exact PCST method and the belief propagation approximation (BP) for $\theta = 0.8$. For $|E| \geq 50$ the PCST method became computationally infeasible for our simulations. The same was true for ASP for $|E| = 150$.

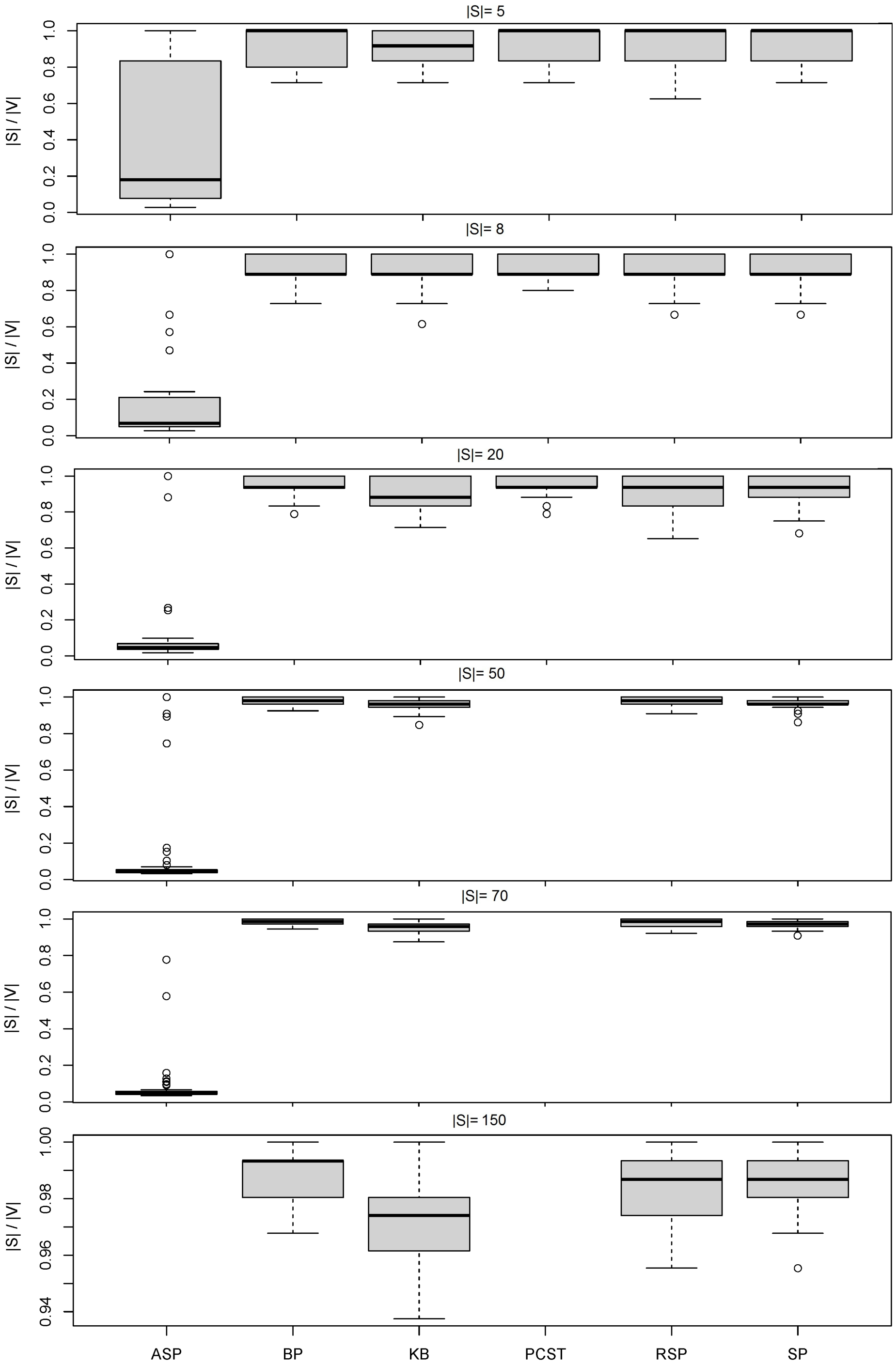

Figure 10: Terminal frequency ($|S|/|V|$) of constructed sub-networks with different heuristic algorithms, the exact PCST method and the belief propagation approximation (BP) for $\theta = 0.8$. For $|E| \geq 50$ the PCST method became computationally infeasible for our simulations. The same was true for ASP for $|E| = 150$.

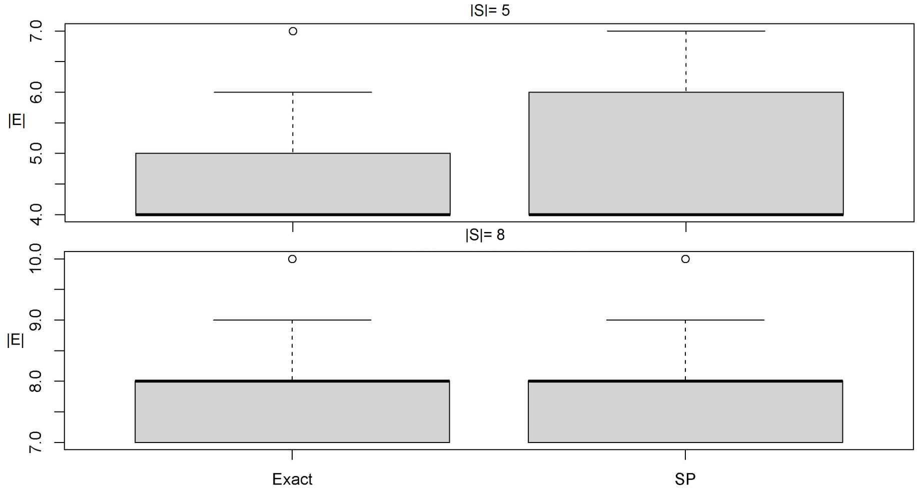

Figure 11: Comparison of the SP approximation to the exact solution: network size ($|E|$) for $\theta = 0.8$.

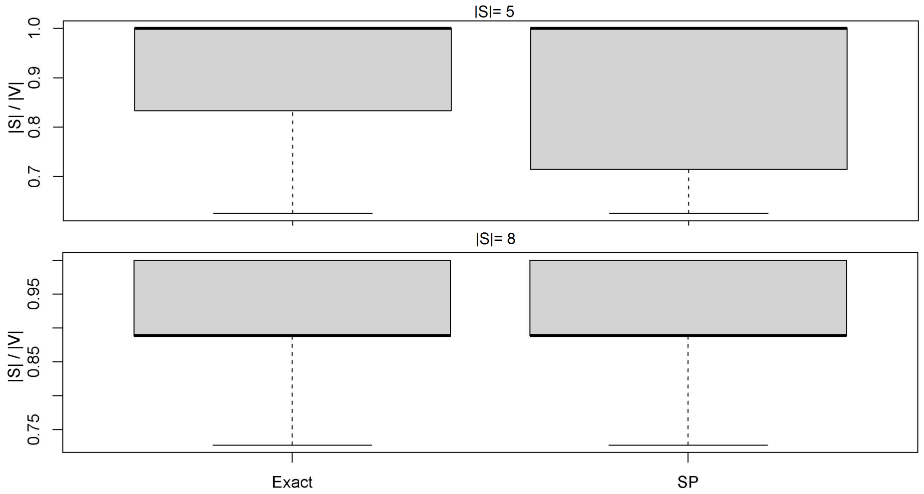

Figure 12: Comparison of the SP approximation to the exact solution: terminal frequency ($|S|/|V|$) for $\theta = 0.8$.

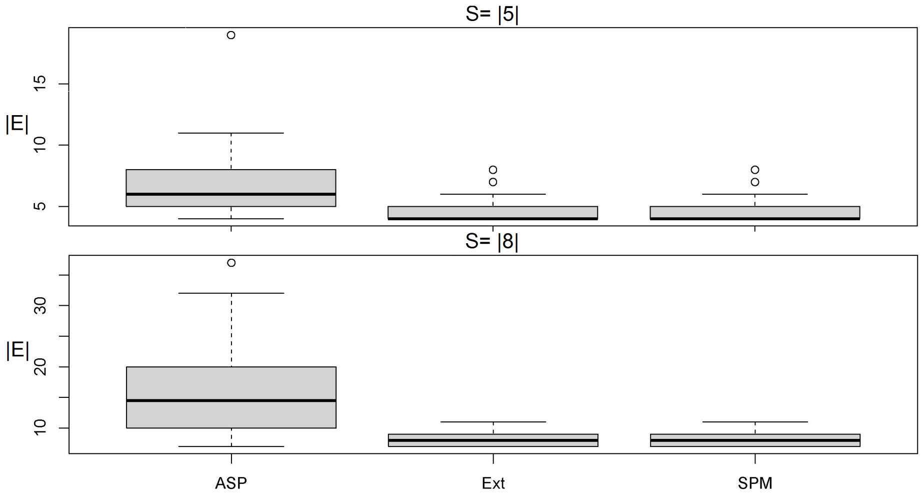

Figure 13: Merged Steiner Trees: Comparison of the ASP and STM approximations to the exact solution in terms of network size ($|E|$) for $\theta = 0.8$.

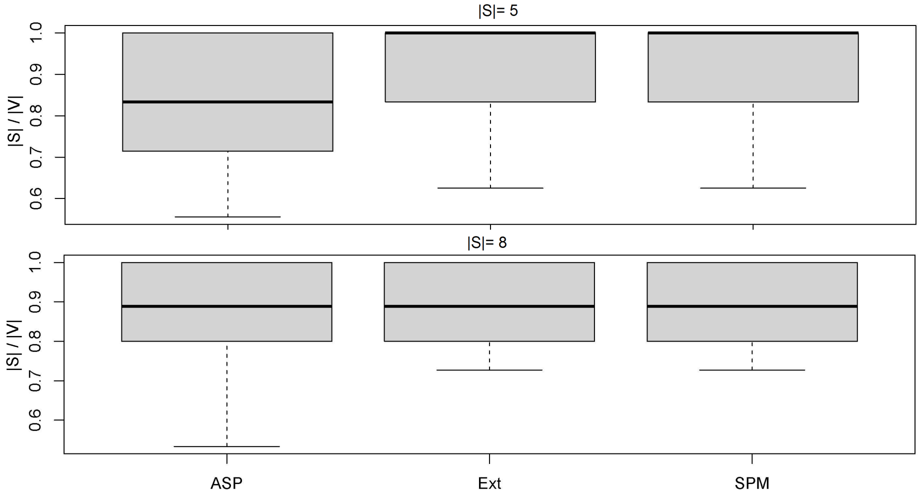

Figure 14: Merged Steiner Trees: Comparison of the ASP and STM approximations to the exact solution in terms of network size ($|E|$) for $\theta = 0.8$.

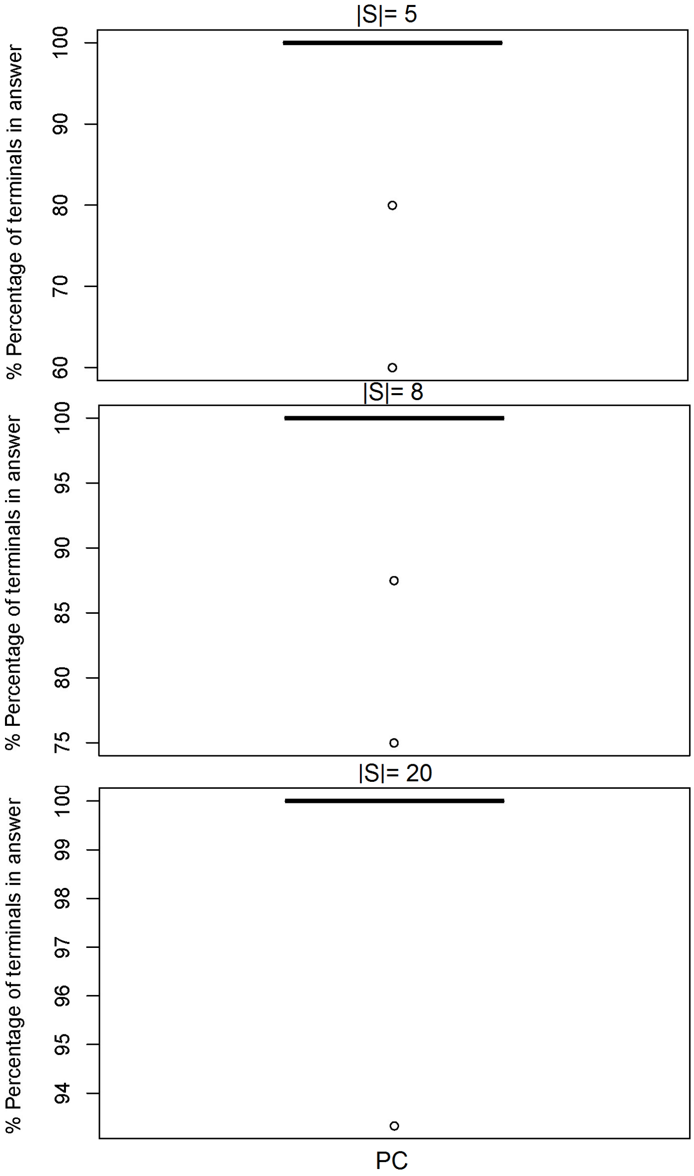

Figure 15: Fraction of terminals recovered by the exact Price Collecting Steiner Tree algorithm for $\theta = 0.8$.

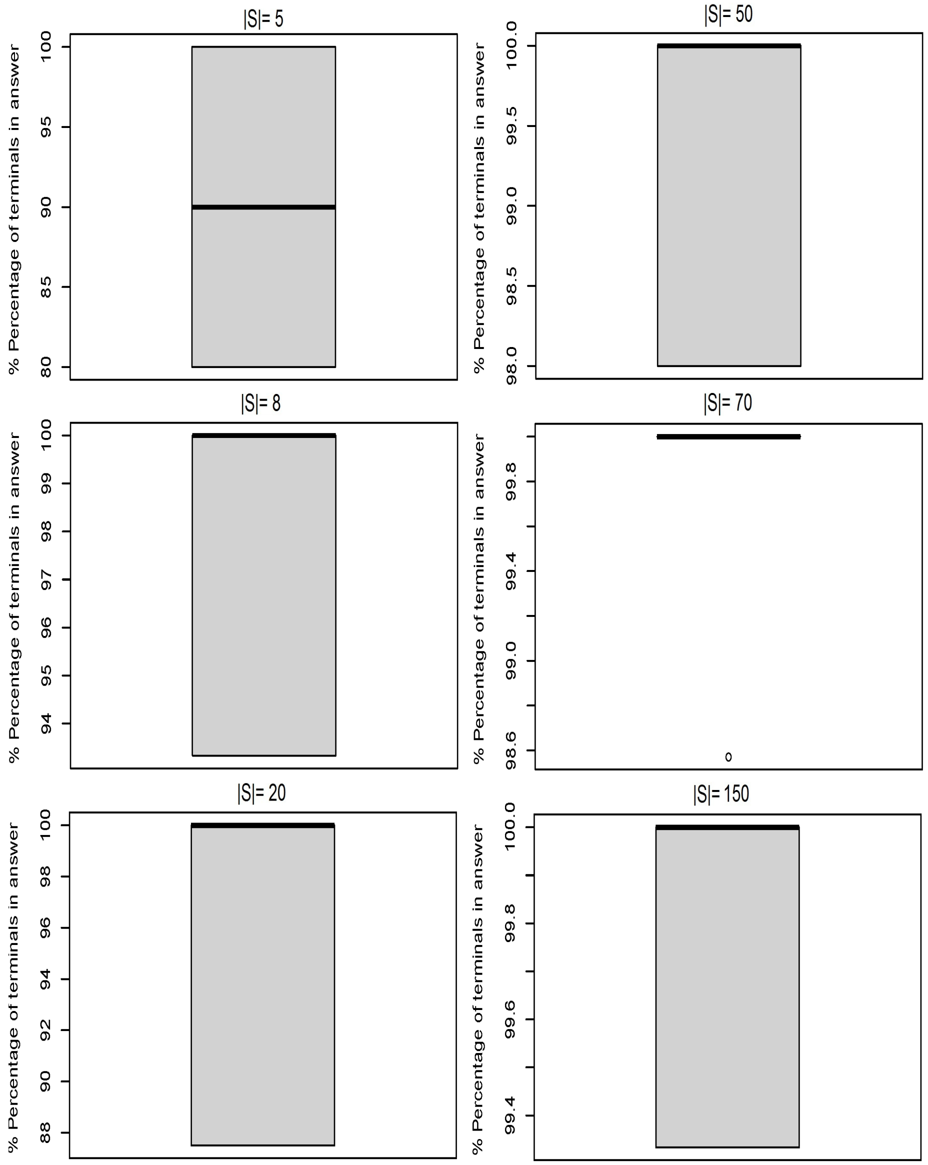

Figure 16: Fraction of terminals recovered by the belief propagation algorithm for $\theta = 0.8$.

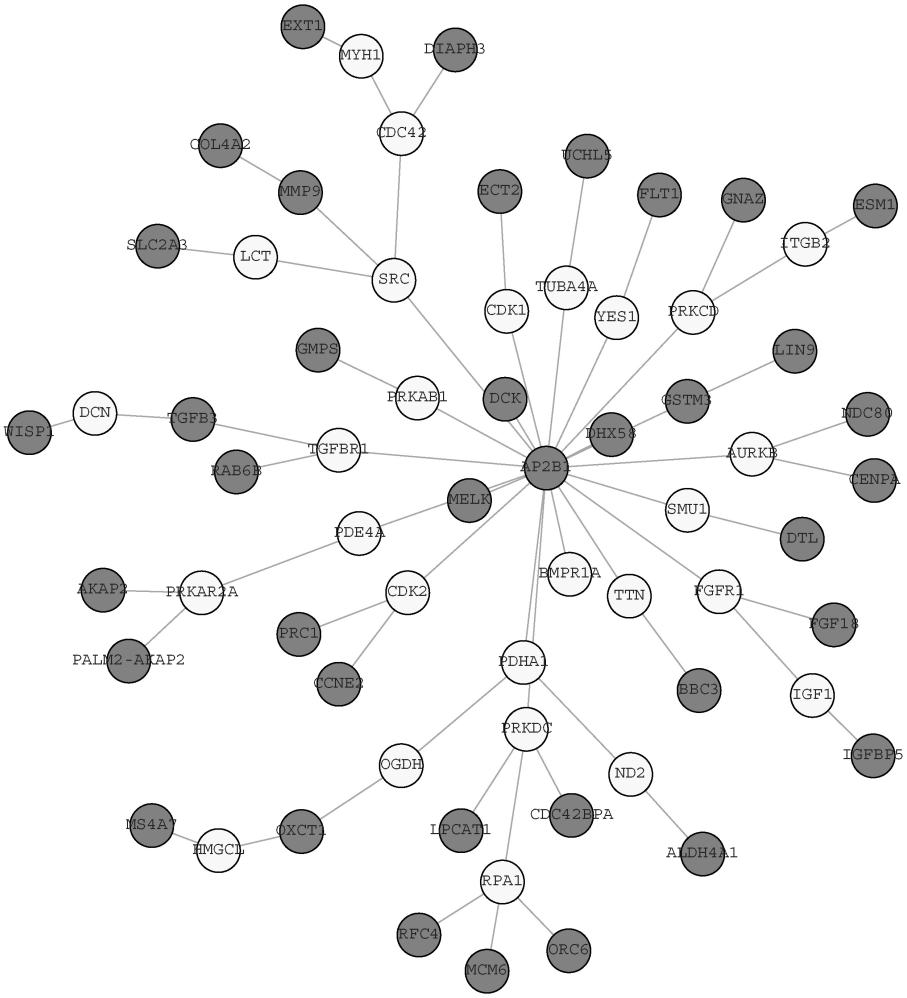

Figure 17: Sub-network reconstructed by the KB algorithm for the prognostic 70 gene breast cancer signature (gray = terminal nodes).

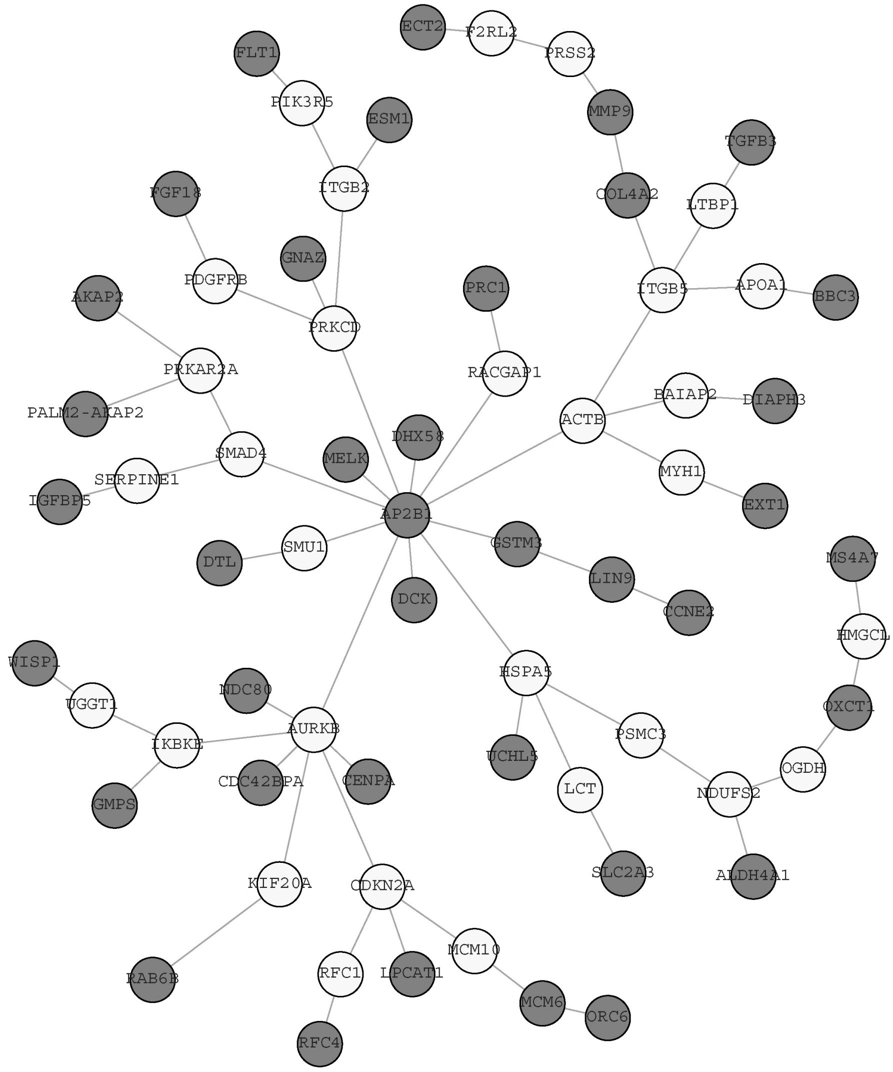

Figure 18: Sub-network reconstructed by the RSP algorithm for the prognostic 70 gene breast cancer signature (gray = terminal nodes).

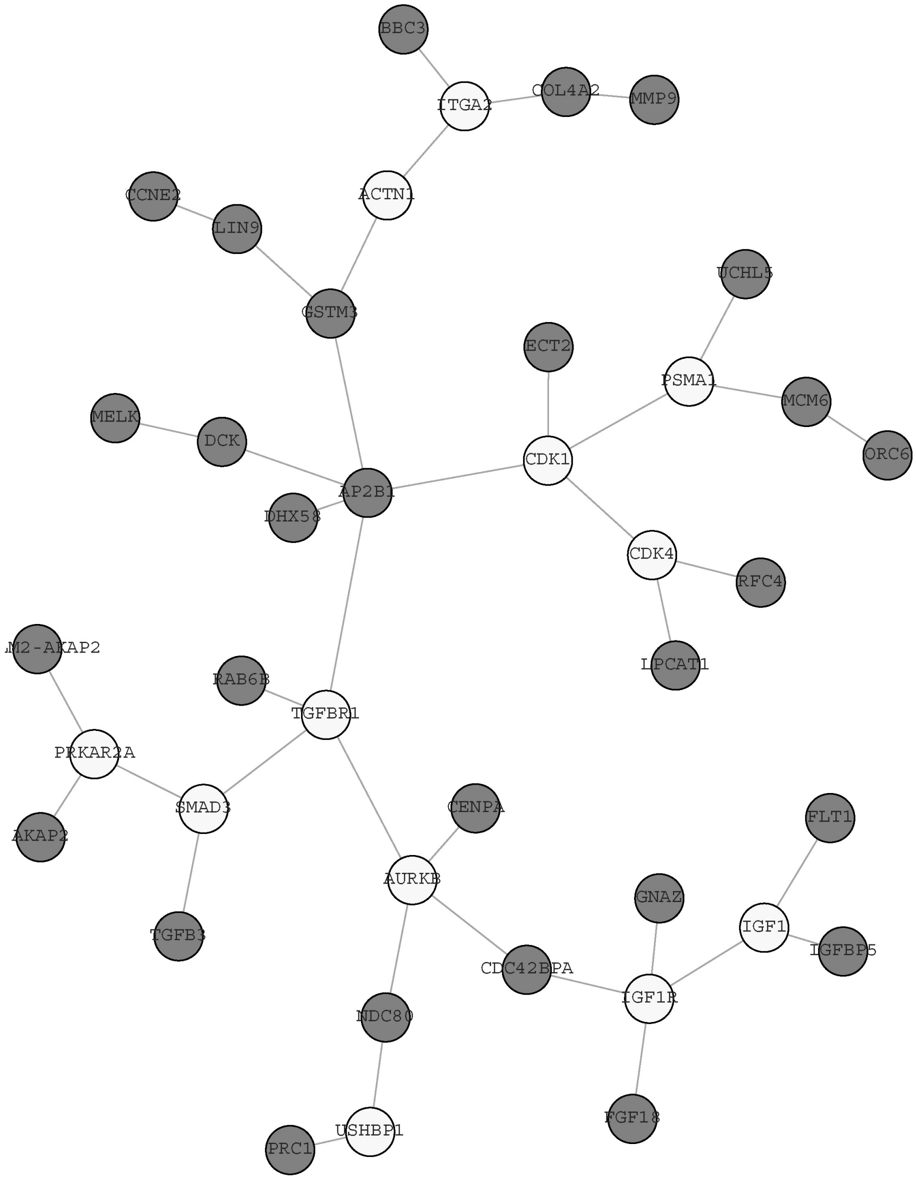

Figure 19: Sub-network reconstructed by the PCST algorithm for the prognostic 70 gene breast cancer signature (gray = terminal nodes). The network contains 28 out of 38 mappable genes.

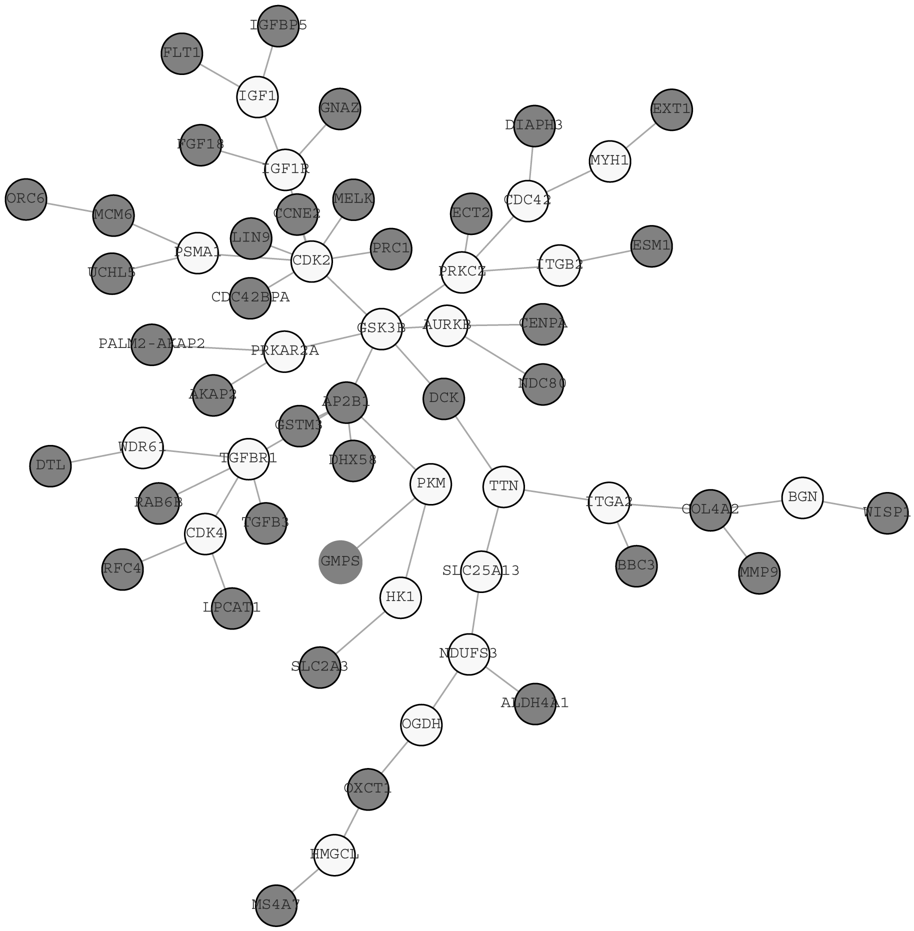

Figure 20: Sub-network reconstructed by the BP algorithm for the prognostic 70 gene breast cancer signature (gray = terminal nodes).

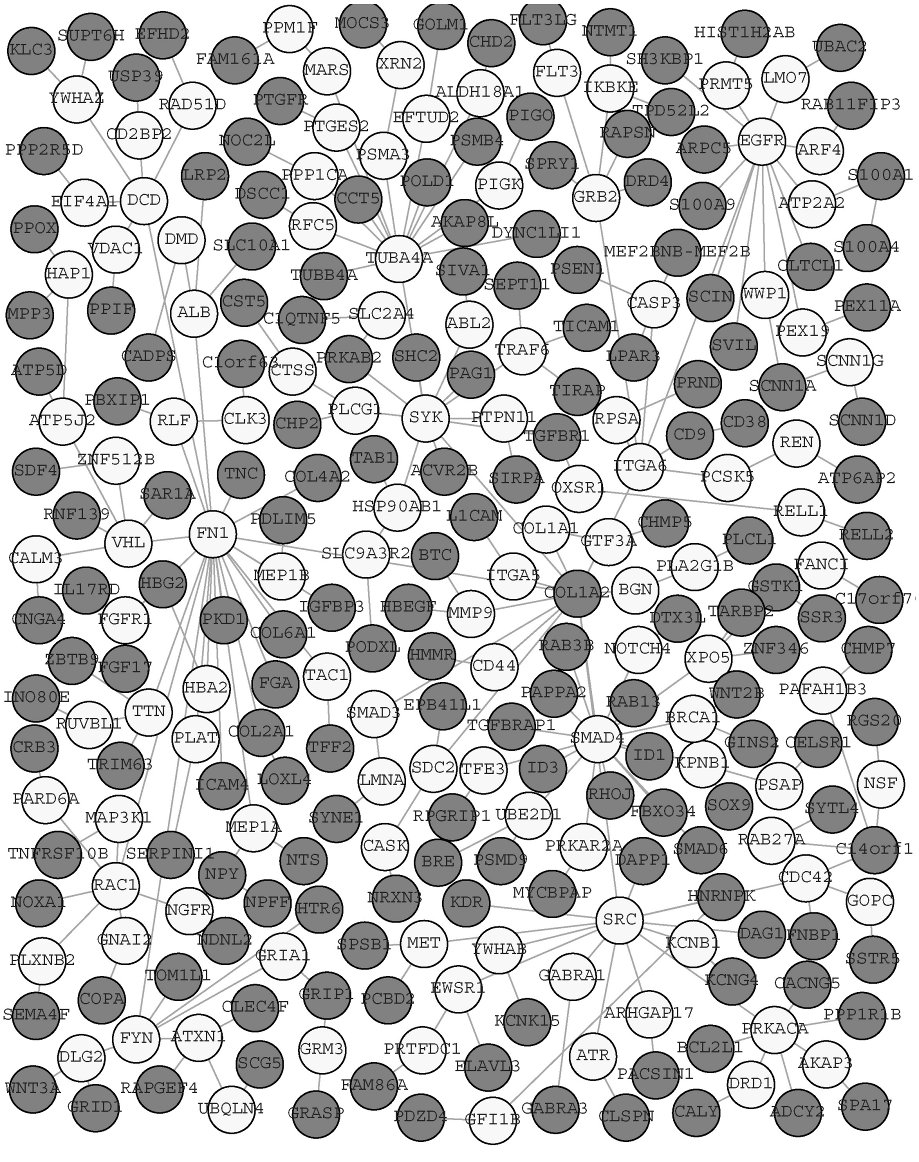

Figure 21: Sub-network reconstructed by the KB algorithm for the 286 gene invasive breast cancer signature (gray = terminal nodes).

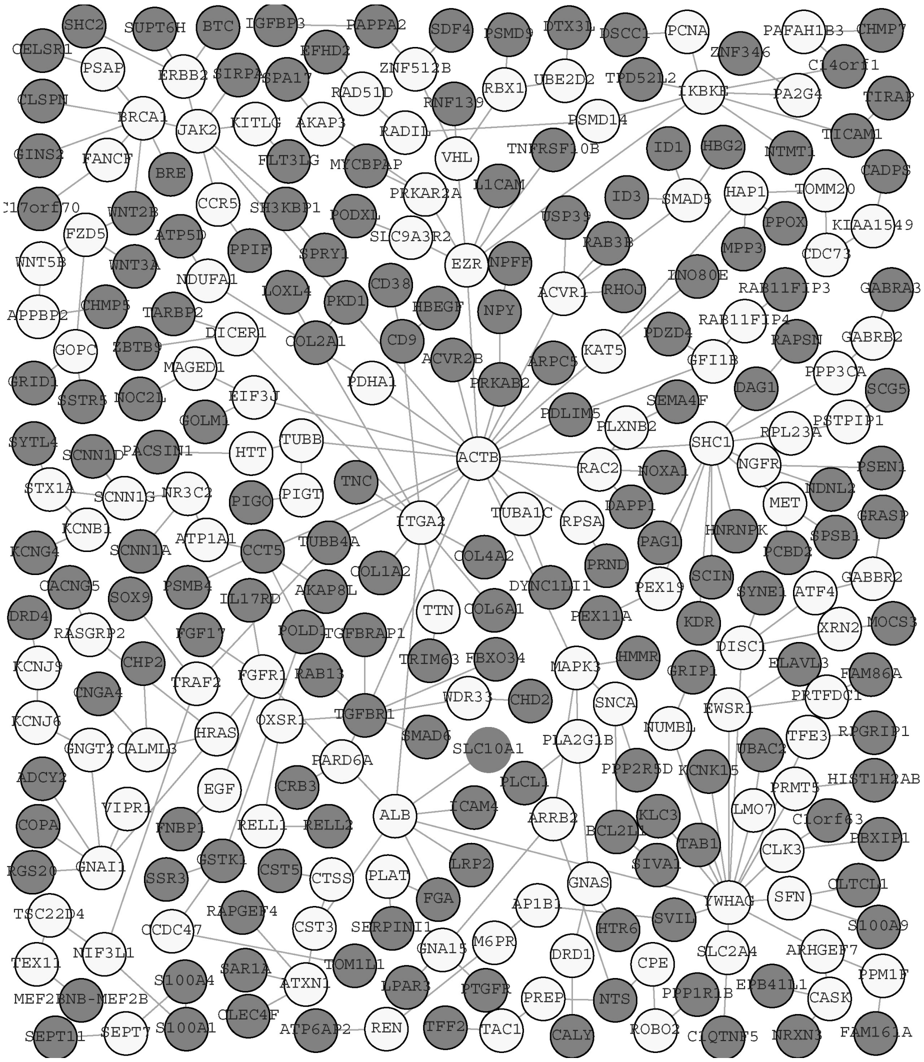

Figure 22: Sub-network reconstructed by the RSP algorithm for the 286 gene invasive breast cancer signature (gray = terminal nodes).

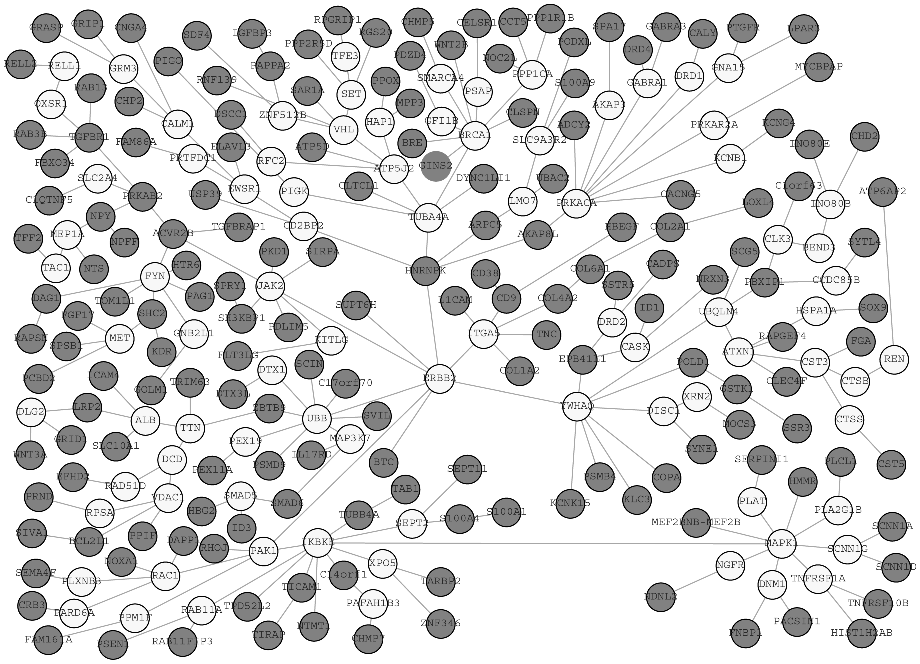

Figure 23: Sub-network reconstructed by the BP algorithm for the 286 gene invasive breast cancer signature (gray = terminal nodes).
