# Supplementary material for: Steiner tree methods for optimal sub-network identification: an empirical study
Source: BMC Bioinformatics. 2013 Apr 30;14:144. doi: 10.1186/1471-2105-14-144 (PMC3674966; doi:10.1186/1471-2105-14-144)
Supplement: Additional file 2 — R-Package SteinerNet. [file 1471-2105-14-144-S2.gz › SteinerNet/inst/doc/SteinerNet.pdf]

Package ‘SteinerNet’

September 20, 2012

Version 1.2

Date 2012-7-22

Title Steiner Tree Approach for Graph Analysis

License GPL-3

Author Afshin Sadegh

Maintainer Afshin Sadeghi <sadeghi.afshin@gmail.com>

Depends R (>= 2.0), igraph0, limma, RBGL

Imports igraph0, limma, RBGL

Description A set of graph functions writen in R to find Steiner trees on graphs. It provides tools for analysing Steiner tree application on networks. It has applications in biological pathway network analysis.

R topics documented:

generate_st_samples 2
steinertree 3
steiner_comparison_plots 5
steiner_simulation 7

Index **9**

generate_st_samples *generate_st_samples*

Description

This function generates simulation data. It creates random graphs with randomly selected terminals.

Usage

```
generate_st_samples(test, graph, folder= NULL, listofterminaltest, repetition)
```

Arguments

- `test` — `test` selects the test type to make random data for it. the random walk for exact algorithm makes subgraphs that include random terminals, but for heuristics it selects random terminals on the base graph and returns the terminal set only
- `graph` — `graph` is the base graph for generating random subgraphs and random terminal set.
- `folder` — `folder` specifies a folder name to store the simulated data inside it.
- `listofterminaltest` — `listofterminaltest` is an input list. Elements of the list are number of terminals to select for a simulation.
- `repetition` — `repetition` is a list of probabilites. Its length declares the number of elements if random data set that is created for terminals. Each element of the list is the probability of selecting a node as terminal while the random walk is traversing the base graph.

Details

This function generates random data for two type of simulations. For experiments that include exact algorithms, it generates random subgraphs with randomly selected terminals. Otherwise it returns only a set of random terminals to be used with the base graph.

Test specifies the type of simulation. `test` can be exact or appr,

`exact` refers to generation of data for an experiment that includes exact Steiner tree algorithm.

`appr` refers to generation of data for a experiment that involves only approximate Steiner tree algorithms.

If `folder` is NULL, it will use default value "steinerdata2" for folder when type is `exact` and "steinerdata" when type is `appr`.

`listofterminaltest` in our study was made of 5, 8, 20, 50, 70 for comparing approximate algorithms and it was 5, 8 for experiments that included exact Steiner tree algorithm. [1]

In our study, we repeated the tests 50 times, and we made the random walk to select a node to be terminal with 0.5 probability. Therefore `repetition` in our comparison was a list of fifty 0.5 values.

Value

The function stores the random data in address that is stated in `folder`. When `test` is exact the output includes random subgraphs and random set of terminals. When `test` is appr the function returns random sets of terminals.

Author(s)

Afshin Sadeghi

References

1. Please refer to the paper that is published with this package.

Examples

```
library(SteinerNet)
g <- graph.ring(10)
#generate_st_samples("exact", g, "testfolder", c(2,3), c(.8,.8))
```

`steinertree` *steinertree*

Description

A function which involves a set of steiner tree algorithms on networks. This set involves an exact algorithm and five heuristic algorithms.

Usage

```
steinertree(type, ter_list = NULL, graph, enumerate = FALSE, coloring = FALSE)
```

Arguments

`type`	`type` specifies which steiner algorithm to use.
`ter_list`	`ter_list` is an input list of terminals. This list should have character type. Steiner tree algorithm finds a solution according to this list.
`graph`	`graph` is an input igraph object which is delivered to one of the steiner tree algorithms of the package. This graph should be connected and it should have undirected edges.
`enumerate`	`enumerate` a boolean value to specify EXA and SPM algorithms. It tells the steiner tree function to return a merged enumerated set of solutions.
`coloring`	`coloring` is a boolean value. When it is TRUE, the function will return two results in a list. The first member of this list represents resulted steiner tree within the input graph by coloring it. In this case the terminals are specified by red color and Steiner nodes are represented by green. Second member of the output list is a single Steiner tree which is represented with a new graph object. When `coloring` is FALSE the function returns the answer in form of a new single graph.

Details

This function withholds six steiner tree algorithms for networks. type specifes the steiner algorithm to deploy to the input graph and terminal set. `type` can be SP, KB, RSP, EXA or SPM. The explanation of the abbreviations is listed below.

`SP` refers to the shortest path heuristic algorithm. [1,2]

`KB` exerts to Kruskal-Based Heuristic algorithm. [3]

`RSP` exerts a random approximation algorithm developed by the package developers. [4]

`EXA` in single mode uses an exact algorithm to return one of the optimal solutions of the problem. In enumerate mode, returns the merged enumerated solution. [4,5]

`SPM` in single mode returns one of heuristic enumeration algorithm solutions for the problem. In enumerate mode, returns the merged enumerated solution.[4]

EXA and SPM algorithms can return a single solution or run in enumerating mode. The boolean value of enumerate specefies one of the two cases. If this value is FALSE they return one of their enumerated steiner solutions without merging it to other solutions. If it is TRUE they return the merged enumerated solutions of the steiner tree problem.

According to our knowledge RSP, EXA Enumeration, SPM and KB are represented for the first time in this package and in the paper that comes with its. [4]

`ter_list` value can be NULL. In this case, the function will observe graph vertex colors to find terminals. To call the function in this approach, the terminal nodes should be colored in red and the non-terminal nodes should be yellow.

This function handles input igraph objects which their vertices have labels and names. To apply the function on graphs with no label and name, `steinertree` function automatically recognizes non-labeled graph vertices and creates names and labels for them. The new labels and names for vertices are created according to the vertice ID of each vertice.

Value

When `coloring` is FALSE returns a Steiner tree in form of a new igraph object. When `coloring` is TRUE returns a list that consists of two objects. The first is a steiner tree and the second object is a colored version of the input graph with distinguished steiner nodes and terminals.

Author(s)

Afshin Sadeghi

References

1. Path heuristic and Original path heuristic ,Section 4.1.3 of the book "The Steiner tree Problem", Petter,L,Hammer

2. "An approximate solution for the Steiner problem in graphs" , H Takahashi, A Matsuyama

3. F K. Hwang, D S. Richards and P Winter,"The steiner tree Problem", Kruskal-Based Heuristic Section 4.1.4,ISBN: 978-0-444-89098-6

4. Please refer to the paper that is pulished with this package.

5. F K. Hwang, D S. Richards and P Winter,"The steiner tree Problem", Kruskal-Based Heuristic Section 4.1.4, The Optimal solution for stiner trees on networks,ISBN: 978-0-444-89098-6.

Examples

```
#example 1
library(SteinerNet)
g <- graph.ring(10)
ter_list= c("1","2","9")
#SP=steinertree("SP", ter_list, g)
#SPM=steinertree("SPM", ter_list, g , TRUE)


#example 2

g2 <- graph( c(0,2,1,2,2,3,3,4,5,6,3,6,2,7,2,5,2,6,5,8), directed=FALSE)
V(g2)$color="yellow"
V(g2)$color[c(1,4)]="red"
OP=steinertree("SP", NULL, g2, FALSE, TRUE)

#example 3: A case study with a sample graph and a given gene list

g <- graph( c(0,2,1,2,2,3,3,4,5,6,3,6,2,7,2,5,2,6,5,8), directed=FALSE)
V(g)$name= c("1058", "51203", "6515", "83879", "160897", "10531", "8659", "2947", "643008")
geneid_list= c("1058","83879", "160897","643")
#we include into the test those geneIDes who exist within the base graph.
r = 1:(length(geneid_list))
t = sapply (r ,function(r) !is.na(match(geneid_list[r],V(g)$name )) )
glist = geneid_list[t==TRUE]
ST1= steinertree( "SP", glist, g)
#tkplot(result1)            # tkplot function displays labels instead of names
```

---

steiner_comparison_plots

steiner_comparison_plots

Description

This function plots the comparison results of Steiner tree algorithms excutions on simulated data.

Usage

```
steiner_comparison_plots (test_name, test_folder =NULL, outputname = NULL, listofterminaltest = NULL ,[illegible]
```

Arguments

`test_name`	`test_name` selects the plot type to creat. 14 type of comparison are available to perform.
`test_folder`	`testfolder` specifies a folder name to read the result of steiner tree simulations.
`outputname`	`outputname` is name of a pdf file to store the result.

listofterminaltest
: listofterminaltest is an input list. Elements of the list are number of terminals that are selected for a simulation.

repetition
: repetition is a list of probabilities. Its length declares the number of elements if random data set that is created for each terminal number.

Details

This function creates 12 different comparison types and depicts them by plots. test_name specifies the type of comparison.

test_name can be on of the following character values.

exact refers to time and edge number comparison of Steiner tree algorithms including the exact algorithm.

appr refers to time and edge number comparison of Steiner tree algorithms without the exact algorithm.

Enum refers to time and edge number comparison of Steiner tree enumeration algorithms.

Enum-median-venn-node-edge refers edge and node number comparison of subgraphs made by Steiner tree enumeration algorithms via Venn diagram.

org refers to edge number comparison of random subgraphs that are made by simulations.

org-dens-e refers to edge density comparison of random graphs that are made by random graph generator.

appr-vfreq refers to vertex frequency comparison of Steiner tree algorithms without the exact algorithm.

exact-vfreq refers to vertex frequency comparison of Steiner tree algorithms including the exact algorithm.

Enum-vfreq refers to vertex frequency comparison of Steiner tree enumeration algorithms.

appr-density-e refers to edge density comparison of steiner tree algorithms excluding the exact algorithm.

exact-density-e refers to edge density comparison of steiner tree algorithms including the exact algorithm.

Enum-density-e refers to edge density comparison of steiner tree enumeration algorithms.

If testfolder is NULL, it will use default value "steinerdatae" for folder when test is exact and "steinerdataenum" when test is enum.

When outputname is NULL, a default value would be used for output pdf file name with consideration of selected type.

listofterminaltest in our study was made of 5, 8, 20, 50, 70 for comparing approximate algorithms and it was 5, 8 for experiments that included exact Steiner tree algorithm. [1]

In our study, we repeated the tests 50 times, and we made the random walk to select a node to be terminal with 0.5 probability while it traverses the base graph. Therefore repetition in our comparison was a list of fifty 0.5 values. If repetition is NULL, the function regards the repetition and listofterminaltest values that were used in our study.

Value

The function stores a resulted plot in a PDF file.

Author(s)

Afshin Sadeghi

References

1. Please refer to the paper that is published with this package.

Examples

```
library(SteinerNet)
g <- graph.ring(10)
#generate_st_samples("exact", g, "testfolder", c(2,3), c(.8,.8))
#steiner_simulation("exact", "testfolder", c(2,3), c(.8,.8))
#steiner_comparison_plots ("exact", "testfolder", c(2,3), c(.8,.8))
```

---

steiner_simulation *steiner_simulation*

Description

This function executes Steiner algorithms on simulated data and stores their results into files.

Usage

```
steiner_simulation(test,listofterminaltest,repetition,testfolder = NULL)
```

Arguments

test	`test` selects the test type to apply the simulation. It can be exact, appr, or enum.
listofterminaltest	`listofterminaltest` is an input list. Elements of the list are number of terminals that are selected for a simulation.
repetition	`repetition` is a list of probabilities. Its length declares the number of elements if random data set that is created for each terminal number.
testfolder	`testfolder` specifies a folder name to read the simulated data from it and to store Steiner tree algorithms results inside it.

Details

This function performs three type of experiments. Test specifies the type of comparison to perform.

`test` can be exact or appr or enum,

`exact` refers to executing the set of steiner tree algorithms including the exact algorithm.

`appr` forces to executing the set of steiner tree algorithms without the exact algorithm.

`enum` refers to to executing the set of steiner tree enumeration algorithms.

`listofterminaltest` in our study was made of 5, 8, 20, 50, 70 for comparing approximate algorithms and it was 5, 8 for experiments that included exact Steiner tree algorithm. [1]

If `testfolder` is NULL, it will use default value "steinerdata2" for folder when `test` is `exact` and "steinerdataenum" when `test` is `enum`.

In our study, we repeated the tests 50 times, and we made the random walk to select a node to be terminal with 0.5 probability. Therefore `repetition` in our comparison was a list of fifty 0.5 values. We also survied the behavior of the algorithms when selection probability was and 0.2 and 0.8.

Value

The function stores the result of execution of Steiner trees and the time of their executions in an address that is stated in `testfolder`.

Author(s)

Afshin Sadeghi

References

1. Please refer to the paper that is published with this package.

Examples

```
library(SteinerNet)
g <- graph.ring(10)
#generate_st_samples("exact", g, "testfolder", c(2,3), c(.8,.8))
#steiner_simulation("exact", "testfolder", c(2,3), c(.8,.8))
```

Index

∗Topic **graphs, protein interaction, network, pathway data graph, steiner tree**
generate_st_samples, 2
steiner_comparison_plots, 5
steiner_simulation, 7
steinertree, 3

generate_st_samples, 2
generate_st_samples, character, igraph object, character, list, list *(generate_st_samples)*, 2
generate_st_samples, character, igraph object, missing, list, list *(generate_st_samples)*, 2

steiner_comparison_plots, 5
steiner_comparison_plots, character, character , character, list, list *(steiner_comparison_plots)*, 5
steiner_comparison_plots, character, character, character, missing, missing *(steiner_comparison_plots)*, 5
steiner_comparison_plots, character, character, missing, list, list *(steiner_comparison_plots)*, 5
steiner_comparison_plots, character, character, missing, missing, missing *(steiner_comparison_plots)*, 5
steiner_comparison_plots, character, missing, missing, missing, missing *(steiner_comparison_plots)*, 5
steiner_simulation, 7
steiner_simulation, list, list, character *(steiner_simulation)*, 7
steiner_simulation, list, list, missing *(steiner_simulation)*, 7
steinertree, 3
steinertree, character list, graph *(steinertree)*, 3
steinertree, character list, graph, boolean *(steinertree)*, 3
steinertree, character list, graph, boolean, boolean *(steinertree)*, 3
steinertree, character list, graph, missing, boolean *(steinertree)*, 3
steinertree, NULL, graph *(steinertree)*, 3
steinertree, NULL, graph, boolean *(steinertree)*, 3
steinertree, NULL, graph, boolean, boolean *(steinertree)*, 3
steinertree, NULL, graph, missing, boolean *(steinertree)*, 3
